# First Partial Skeleton of a 1.34-Million-Year-Old *Paranthropus boisei* from Bed II, Olduvai Gorge, Tanzania

**DOI:** 10.1371/journal.pone.0080347

**Published:** 2013-12-05

**Authors:** Manuel Domínguez-Rodrigo, Travis Rayne Pickering, Enrique Baquedano, Audax Mabulla, Darren F. Mark, Charles Musiba, Henry T. Bunn, David Uribelarrea, Victoria Smith, Fernando Diez-Martin, Alfredo Pérez-González, Policarpo Sánchez, Manuel Santonja, Doris Barboni, Agness Gidna, Gail Ashley, José Yravedra, Jason L. Heaton, Maria Carmen Arriaza

**Affiliations:** 1 Instituto de Evolución en África, Museo de los Orígenes, Madrid, Spain; 2 Department of Prehistory, Complutense University, Madrid, Spain; 3 Department of Anthropology, University of Wisconsin-Madison, Madison, Wisconsin, United States of America; 4 Evolutionary Studies Institute, University of the Witwatersrand, Johannesburg, South Africa; 5 Plio-Pleistocene Palaeontology Section, Department of Vertebrates, Ditsong National Museum of Natural History (Transvaal Museum), Pretoria, South Africa; 6 Museo Arqueológico Regional, Plaza de las Bernardas s/n, Madrid, Spain; 7 Archaeology Unit, University of Dar es Salaam, Dar es Salaam, Tanzania; 8 Natural Environment Research Council Argon Isotope Facility, Scottish Universities Environmental Research Centre, East Kilbride, Scotland, United Kingdom; 9 Department of Anthropology, University of Colorado Denver, Denver, Colorado, United States of America; 10 Department of Geodynamics, Complutense University, Madrid, Spain; 11 Research Laboratory for Archaeology and the History of Art, University of Oxford, Oxford, United Kingdom; 12 Department of Prehistory and Archaeology, University of Valladolid, Valladolid, Spain; 13 Centro Nacional de Investigación sobre la Evolución Humana, Burgos, Spain; 14 Centre Européen de Recherche et d'enseignement de Géosciences de l'Environnement, Aix-Marseille Université, Aix-en-Provence, France; 15 Paleontology Unit, National Museum of Tanzania, Dar es Salaam, Tanzania; 16 Department of Earth and Planetary Sciences, Rutgers University, Piscataway, New Jersey, United States of America; 17 Department of Biology, Birmingham-Southern College, Birmingham, Alabama, United States of America; University of New South Wales, Australia

## Abstract

Recent excavations in Level 4 at BK (Bed II, Olduvai Gorge, Tanzania) have yielded nine hominin teeth, a distal humerus fragment, a proximal radius with much of its shaft, a femur shaft, and a tibia shaft fragment (cataloged collectively as OH 80). Those elements identified more specifically than to simply Hominidae gen. et sp. indet are attributed to *Paranthropus boisei*. Before this study, incontrovertible *P. boisei* partial skeletons, for which postcranial remains occurred in association with taxonomically diagnostic craniodental remains, were unknown. Thus, OH 80 stands as the first unambiguous, dentally associated *Paranthropus* partial skeleton from East Africa. The morphology and size of its constituent parts suggest that the fossils derived from an extremely robust individual who, at 1.338±0.024 Ma (1 sigma), represents one of the most recent occurrences of *Paranthropus* before its extinction in East Africa.

## Introduction

Apart from the occurrence of articulating bones from the same skeleton, the close intrasite vertical (i.e., stratigraphic) and horizontal (i.e., lateral) spatial association of isolated hominin postcranial bone specimens and taxonomically diagnostic craniodental remains is the most secure (but not definitive) way that paleoanthropologists can begin to describe and contrast inter-generic and inter-specific adaptations of the hominin postcranium. Currently, there are secure or fairly secure craniodental-postcranial associations (thus, they meet at least one of the criteria above) for six early Neogene hominin species: *Ardipithecus ramidus* [1]; *Australopithecus afarensis* [2]; *Au.* sp. [3]; *Au. sediba* [4]; *Homo habilis* [5] and *H. ergaster* [6]. The status of *Paranthropus boisei*, a craniodentally highly derived Pleistocene species, is less clear because we know, based on fossil evidence from various stratigraphic intervals and sites in East Africa, that it co-existed with early *Homo* for a significant interval [7]. For instance, KNM-ER 1500, a fragmentary hominin postcranium from Koobi Fora (Kenya), was assigned to *P. boisei* based on its surficial spatial association with a mandible fragment that has a thick corpus and marginal crest [8]. Wood [9] noted, however, that some penecontemporaneous, dentally-identified mandibles of early *Homo* show the very same traits, calling into question the taxonomic attribution of KNM-ER 1500 to *P. boisei*. Wood and Constantino [7] went further, reviewing all claims of *P. boisei* postcranial remains, and deeming each dubious for a variety of enumerated reasons.

Here we describe hominin dental and postcranial remains (cataloged collectively as OH 80) that were recovered *in situ* in close spatiotemporal proximity through excavations in Level 4 of the BK (Bell's Korongo) site, in Upper Bed II, Olduvai Gorge (Tanzania). Based on diagnostic morphological criteria, the most complete BK tooth fragments are allocated confidently to *P. boisei*. There are no replicated skeletal elements in the BK Level 4 hominin sample, the state of preservation all of hominin fossils is consistent and they all derive from a circumscribed horizontal area (see SI Appendix). In addition, the BK faunal assemblages, overall, show limited evidence, in the form of bone breakage patterns and tooth mark damage, of significant carnivore involvement in their formations [10]. However, all four of the Level 4 hominin postcranial fossils are broken in patterns consistent with static loading of green bone, such as is accomplished by carnivore chewing (e.g., [11]). Based on taphonomic parsimony, these lines of evidence support the hypothesis that all of the Level 4 hominin fossils derive from a single individual, and, based on taxonomically diagnostic characteristics of the teeth, that that individual represents the species *P. boisei*.

## Results and Discussion

### Site age and summary

The BK site is situated in Bed II of Olduvai Gorge, directly above tuff IID, which was previously dated at c. 1.2 Ma [12], [13]. We have determined a new ^40^Ar/^39^Ar age for tuff IID at BK of 1.353±0.035 Ma (1 sigma, full external precision, Renne et al. [14], see Appendix S1). Tuff IID has also been identified as a primary tuff at RHC (Richard Hay Cliff) [13], and we have confirmed this correlation with an age of 1.321±0.032 Ma (1 sigma, full external precision, Renne et al. [14], see Figures S10 and S11 and Table S6 in Appendix S1). Taking an average of these two age constraints, we report the age for tuff IID of Olduvai Gorge as 1.338±0.024 Ma. This age is discussed throughout the remainder of this contribution. Hay [13] also suggested a correlation of tuff IID at BK and RHC with exposed tuffs at JK (Juma Korongo) and MCK (Margaret Cropper Korongo). New ^40^Ar/^39^Ar age data (see Appendix S1) show these two tuffs to be 1.992±0.009 and 1.975±0.029 Ma (1 sigma, full external precision, Renne et al. [14], see Appendix S1), respectively, showing such a correlation to be inaccurate. We also attempted to correlate between all sites using glass chemistry but data were equivocal (see Appendix S1).

The hominin-bearing site consists of fluvial deposits eroding into tuff IID and its overlying clay and tufa. To date, a sequence of eight discrete archaeological levels are recognized within a fluvial alluvial deposit (see Appendix S1). The OH 80 hominin fossils were excavated from Level 4, which is characterized by a large assemblage of Mode I stone tools that are associated functionally with abundant vertebrate fossils. Butchery marks link the tools and fossils causally, and a preponderance of the butchery damage occurs on fossils of ungulates of medium, large and very large body sizes [10]. The BK zooarchaeological assemblages [10], [15]–[17], along with those from FLK 22 *Zinjanthropus* (Bed I, Olduvai) and Peninj (Tanzania), constitute the best evidence of hominin butchery and meat-eating in the early Pleistocene.

### Dental fossils (Figure 1; Table 1)

**Figure 1:**
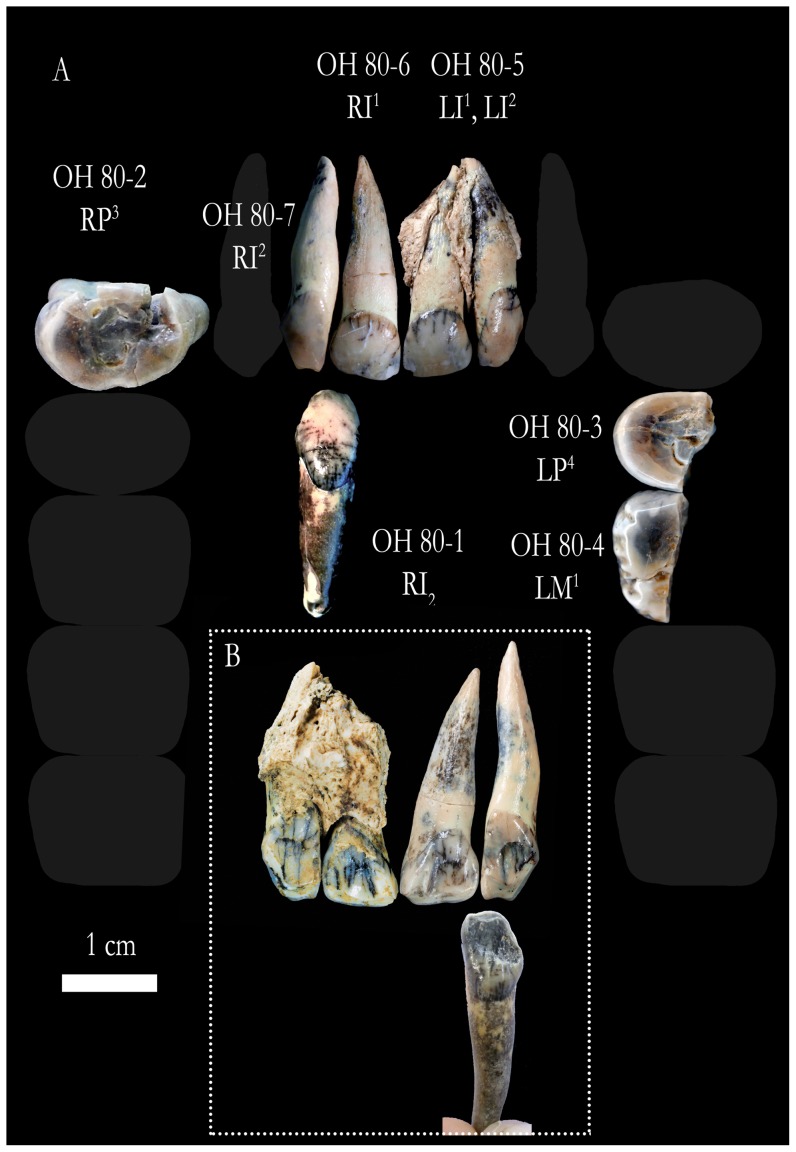
Hominin dental remains from Level 4 at the BK site. Teeth are shown in approximate anatomical position on a schematic dental arcade; anterior teeth are shown in labial view, postcanine teeth in occlusal view (A). Labial views of the anterior teeth are shown in (B). Photographs of individual teeth by J Trueba and MJ Ortega; composition by JL Heaton.

**Table 1:** Comparative metric analysis of the OH80 dentition and the *Paranthropus boisei* available sample (obtained from the reference 9 and the Human Origins Data Base: http://humanoriginsdatabase.org), including OH5.

tooth	specimen	BL	MD
upper I1	OH80-5	6.8	7.8
upper I1	OH80-6	6.8	7.8
upper I1	OH5	8	>10
upper I1	Pb* mean (range)	7(6.1–8)	8.8(8–>10)
upper I2	OH805	7.8	6.1
upper I2	OH80-7	7.9	6.1
upper I2	OH5	7.5	6.9
upper I2	Pb* mean (range)	6.4(5.6–7.5)	6.5(6.2–6.9)
upper P3	OH80-2	16.2	10.4
upper P3	OH5	17	10.9
upper P3	Pb* mean (range)	15.2(14.2–17)	10.8(10.2–12)
upper P4	OH80-3	-	10.5
upper P4	OH5	(17.6–18)	12
upper P4	Pb* mean (range)	15.8(12.2–18)	11.4(9.1–12.5)
upper M1	OH80-4	-	14.2
upper M1	OH5	17.7	15.2
upper M1	Pb* mean (range)	16.2(14.9–17.7)	14.8(13.5–15.7)
lower I2	OH80-1	6.6	6.2
lower I2	Pb* mean (range)	6.9(6.4–8.1)	6.4(6.1–6.6)

* Paranthropus boisei.

Data for *Paranthropus boisei* include mean values plus range (minimum and maximum). Measurements are in mm.

OH 80-1 is a nearly complete RI_2_, with just a small chip from the apex on the labial surface of its root. The lingual surface of the crown possesses two prominent marginal ridges converging onto its gingival eminence, which projects strongly distally and shows a bulging tubercle crossed by deep mesial and distal grooves, with other secondary grooves converging on it. The marginal ridges demarcate a lingual fossa, which is most prominent distally. The crown's incisal surface shows flat wear, which extends onto its distal edge sloping cervico-distally, giving the crown strong asymmetry in labial and lingual outline. The enamel bulges just superior to the clearly demarcated cervicoenamel junction (CEJ). The root expands just inferior to the crown and tapers very gently to a deflected tip. Measurements of the mesiodistal (MD) diameters for this tooth and the upper lateral incisors (see below) have been corrected for interstitial wear. Overall, the tooth is very canine-like in form, but its exceedingly small size (MD = 6.2 mm; LL = 6.6 mm) and good occlusion with OH 80-7, a RI^2^ described below, are the bases of its allocation as a lower lateral incisor. The mean MD and LL diameters for *P. boisei* I_2_s are, respectively, 6.4 mm (sd = 0.22; n = 6) and 6.9 mm (sd = 0.79; n = 4), versus C_1_ mean values of 7.7 mm (sd = 0.92; n = 10) (MD) and 8.7 mm (sd = 0.84; n = 11) (LL) (calculated from data in ref. [9]). Together, the morphology and diminutiveness of OH 80-1 justify its allocation to *P. boisei*.

OH 80-2 is a fragmentary RP^3^ attached to a small fragment of maxilla. The maxilla segment is well preserved, showing three partial alveoli, but is otherwise uninformative. The tooth is composed of a nearly complete crown, two buccal roots and one lingual root, which are all clearly separated. Mesially, the crown is missing a portion of its lingual cusp as part of a complicated postmortem fracture that assumes the form of a narrow channel occlusally (45 mm in MD length), and then carries onto the mesial face, where it expands buccolingually at the approximate location of the CEJ. Where undamaged, the CEJ on the lingual face is roughly horizontal superior to a prominent cingulum bulge, typical of *Paranthropus* premolars. On the buccal face of OH 80-2, the enamel bulges inferior to its well-rounded CEJ. There is an oval interproximal wear facet (1.7 mm buccolingually) on the distal side of the crown due to contact with the P^4^. The occlusal surface of the crown is moderately worn in a flat pattern, but a mesio-distal groove, separating the buccal and lingual cusps, is still present; the enamel of the crown's distal shoulder shows a slight inflection at the distal terminus of the mesial-distal groove. Remnants of the mesial and distal fossae are also apparent. In occlusal outline, the crown is oval and possesses two low shoulders mesio- and distobuccally, giving OH 80-2 a molariform shape, typical of *P. boisei* [18], [19]. The crown of OH 80-2 (BL = 16.2 mm; MD = 10.4 mm) is slightly smaller than are the crowns of the P^3^s of the *P. boisei* cranium OH 5 (BL = 17.0 mm; MD = 10.9 mm) [18].

OH 80-3 is LP^4^ fragment that preserves the lingual portion of its crown and the lingual root, save its apex. The superior portion(s) of the buccal root(s) was/were broken postmortem and is/are missing, but its/their inferior portion(s) connect(s) to the lingual root at slightly less than one third of the lingual root's full length. The preserved crown portion of OH 80-3 (MD = 10.5 mm) is composed mostly of the lingual cusp and well-worn remnants of the mesial-distal groove and distal fossa; the specimen is truncated by a postmortem fracture edge just buccal to these occlusal features. The occlusal surface is worn flat and to the same degree as is OH 80-2, but still maintains a thick enamel cap. Enamel thickness along the natural mesiodistal section ranges from 1.6 to 1.7 mm. Collectively, these observations prompt our assignment of OH 80-3 to *P. boisei* and to the same individual from which OH 80-1 and OH 80-2 are inferred to have derived.

OH80-4 is the partial protocone and hypocone of a LM^1^, with an attached lingual root fragment. Because the specimen was naturally fractured postmortem along a mesiodistal plane, it allows measurement of the enamel cap thickness, which is 2.5 mm at the protocone and 2.9 mm at the hypocone. The crown's occlusal surface is moderately worn (slightly less than are those of the OH 80 premolars) but retains evidence of moderate crenulation and a prominent lingual groove, which continues strongly onto the crown's lingual face. The lingual face of the tooth's protocone is traversed by a series of superoinferior trending grooves, representing a complex Carabelli's formation, comparable in intensity to those on the maxillary molars of OH 5. The lingual enamel bulges just inferior to the horizontally running CEJ. The 14.2 mm MD diameter of OH 80-4 is 1 mm shorter than are the MD diameters of the M^1^s of OH 5 [18]. The preserved lingual root is short (17.4 mm superoinferiorly). Together, these metric and morphological observations (in addition to spatial proximity) prompt the assignment of OH 80-4 to *P. boisei* and to the same individual as represented by OH 80-1, 2 and 3.

OH 80-5 is a 5 cm mesiodistally wide fragment of left maxilla with its implanted I^1^ and I^2^. The incisors' alveoli are nearly complete lingually but largely broken away labially. Part of the mesial wall of the alveolus for the RI^1^ is preserved, in which OH 80-6 (described below) fits perfectly. The crown of the OH 80-5 I^1^ is altered diagenetically, with several vertical cracks emanating from the CEJ and with geochemically induced staining (in the form of white circular patches and lines) on its labial face, near the incisal margin. Fine striations, which might be the result of *in vivo* wear and/or postmortem movement of the specimen in abrasive sediments, are also observed on both faces of the tooth's crown. The crown is moderately worn, with the incisal margin curving gently onto the distal corner and, showing a height of 8.4 mm, measured lingually. The crown is also smaller (MD = 7.8 mm; BL = 6.8 mm) than the measurable I^1^ of OH 5 (MD>10 mm; BL = 8.0 mm) [18], but accords with the I^1^ mean values for the *P. boisei* hypodigm (MD = 8.8 mm [sd = 0.93; n = 4]; BL = 7.0 mm [sd = 0.77; n = 5]) (calculated from data in ref. [9]). The lingual surface is slightly shoveled, showing two well-developed marginal ridges that converge on the moderately developed cingulum bulge. This LI^1^ is the antimere of OH 80-6, which, like the OH 80-5 LI^1^ is diagenetically discolored in patches and shows nearly identical morphology and dimensions (MD = 7.8 mm; BL = 6.8 mm).

The OH 80-5 LI^2^ is the antimere of OH 80-7. OH 80-7 occludes with OH 80-1, the RI_2_ described above. Like OH 80-1, each of the upper lateral incisors shows distinct, canine-like morphology, evincing strong asymmetry in labial and lingual view and has marked incisal wear that tapers distally, reaching nearly the incisal extent of the cingulum bulge. Fortunately, even though the mesial face of the RP^3^ OH 80-2 is damaged (see above), enough of that tooth's mesial interproximal wear facet is preserved to demonstrate that OH 80-7 does not match it, as should be the case if both teeth derived from the same individual and if OH 80-7 was a RC^1^ and not a RI^2^. The MD diameters of the OH 80-5 I^2^ and OH 80-7 are both 6.1 mm; their LL diameters are, respectively, 7.8 mm and 7.9 mm. These values match more closely the mean values of *P. boisei* I^2^s (MD = 6.5 mm [sd = 0.35; n = 5]; BL = 6.6 mm [sd = 0.91; n = 6]) than the mean values of *P. boisei* C^1^s (MD = 8.8 mm [sd = 0.80; n = 8]; BL = 9.0 mm [sd = 0.86; n = 7]) (calculated from data in ref. [9]). The crowns of both upper lateral incisors are slightly shoveled, with moderately developed marginal ridges and gingival eminences. We note that the morphology of the OH 80 upper lateral incisors is significantly different than that of the upper lateral incisors of OH 5, the latter of which are more similar to typical central incisors in shape and apical wear. OH 80-5 and OH 80-7, in contrast, show interstitial wear, as a result of their more canine-like morphology. Given the similarities between the other teeth of OH 80 and those of the *P. boisei* hypodigm, the most parsimonious approach is to consider the differences in lateral incisor morphology as simply demonstrating a large range of variation in *P. boisei*, It is also worth noting that the OH 5 *P. boisei* holotype is half a million years older than OH 80, so diachronic variation in the two fossils should not be unexpected. However, if new fossil discoveries from OH 80's interval prove to show even more differences with the *P. boisei* hypodigm, then the taxonomic status of OH 80 might, at that time, need to be re-evaluated.

OH 80-8 (MD = 8.6 mm; superoinferior = 13 mm) and OH 80-9 (MD = 8.9 mm; superorinferior = 15.7 mm) (not figured) are two extremely robust molar roots missing their crowns and deriving from teeth other than those previously described. There is, however, no reason to infer that they represent a species other than *P. boisei* or an individual other than that inferred to have provided the previously described dental fossils.

The OH 80 dental fossils are permanent teeth, representing a single adult *P. boisei* (with small anterior teeth and large postcanine teeth that are worn flat, and includes a molariform premolar—a character suite typical of the species [18], [19]), and differentiated from the juvenile *P. boisei* represented by OH 3, a deciduous canine and molar set, discovered previously at BK [20]. Based on our new ^40^Ar/^39^Ar ages for tuff IID, the presence of OH 3 and OH 80 at the site provides a new last appearance datum for *P. boisei*, shifting that datum from c. 1.4 Ma (Konso Formation, Ethiopia; ref. [21]) to younger than 1.338±0.024 Ma.

### Humerus

OH 80-10 is a distal portion of a left humerus diaphysis, measuring 88.0 mm in maximum length. The specimen terminates at both ends in green bone fractures and, although no tooth marks are preserved on its periosteal surface, this type of fracture pattern is consistent with damage imparted on long bones by feeding carnivores (e.g., [11]). Its surface is well preserved, with no subaerial weathering (0 in Behrensmeyer's [22] bone weathering stage system) but some manganese dioxide staining on its anterior aspect. Additionally, part of the medial supracondylar crest was removed by diagenetic chemical alteration. The spiral fracture that terminates the specimen proximally is inferior to the nutrient foramen and medial crest. Cortical bone, as measured perio-endosteally, at the proximal fracture is 7.0 mm, considerably thicker than in modern *H. sapiens* [23] (Figures S1 and S2 in Appendix S1). This remarkable cortical robusticity is consistent with the hypothesis that OH 80-10 derives from the same large individual as is represented by the radius specimen, OH 80-11, described below. The distal fracture edge of OH 80-10 is through the superior portion of the supracondylar ridges, with the dorsal periosteal surface in that position presenting a shallow concavity that is the superior-most portion of the otherwise missing olecranon fossa. The distal fracture edge of OH 80-10 is close enough anatomically to the transverse line at which Susman et al. [24] made CT-scan sections on four fossil hominin humeri from Swartkrans Cave (South Africa) that comparisons are possible. The shape of the distal portion of OH 80-10 is broad in ML dimension and flat in AP dimension. This is a similar configuration as that of the inferred Swartkrans *P. robustus* humeral specimens SKX 19495, SK 24600 and SK 2598 [24]. Additionally, like other inferred *Paranthropus* humerus specimens, the distal diaphyseal section of OH 80-10 is, overall, more rounded than the triangular humerus diaphyseal sections of *Homo* [23]. Mediolateral shaft thicknesses taken at the superior margins of the olecranon fossae in SKX 19495, SK 24600 and SK 2598 (range = 28.7–31.6 mm) also accord with that of OH 80-10 (30 mm, a probable underestimate due to the incompleteness of the supracondylar ridges). In contrast, AP thickness in this region is greater for OH 80-10 (15.6 mm) than are those of the Swartkrans specimens (range = 11.1–12.5 mm). The inter-specimen discrepancy in AP thickness might be due to any number of factors, including the possibility that SKX 19495 and SK 24600 are derived from small (female?) individuals [24] and that OH 80-10 is derived from a large (male?) individual. Morphologically, the shaft section shape of OH 80-10 is very similar to SK 24600, with both specimens displaying elongated ML planes and slight concavities anteriorly (remembering, though, that the OH 80-10 section is situated superior to the bicondylar line). In sum, the preserved morphology of OH 80-10 contrasts with that known for definitive early *Homo* humeri and comports with that of inferred *Paranthropus* humeri from Swartkrans. The possibility exists that the Swartkrans specimens in question do not actually represent *Paranthropus*, but currently their allocation, and that of OH 80-10, to that genus is the most parsimonious hypothesis.

### Radius

**Figure 2:**
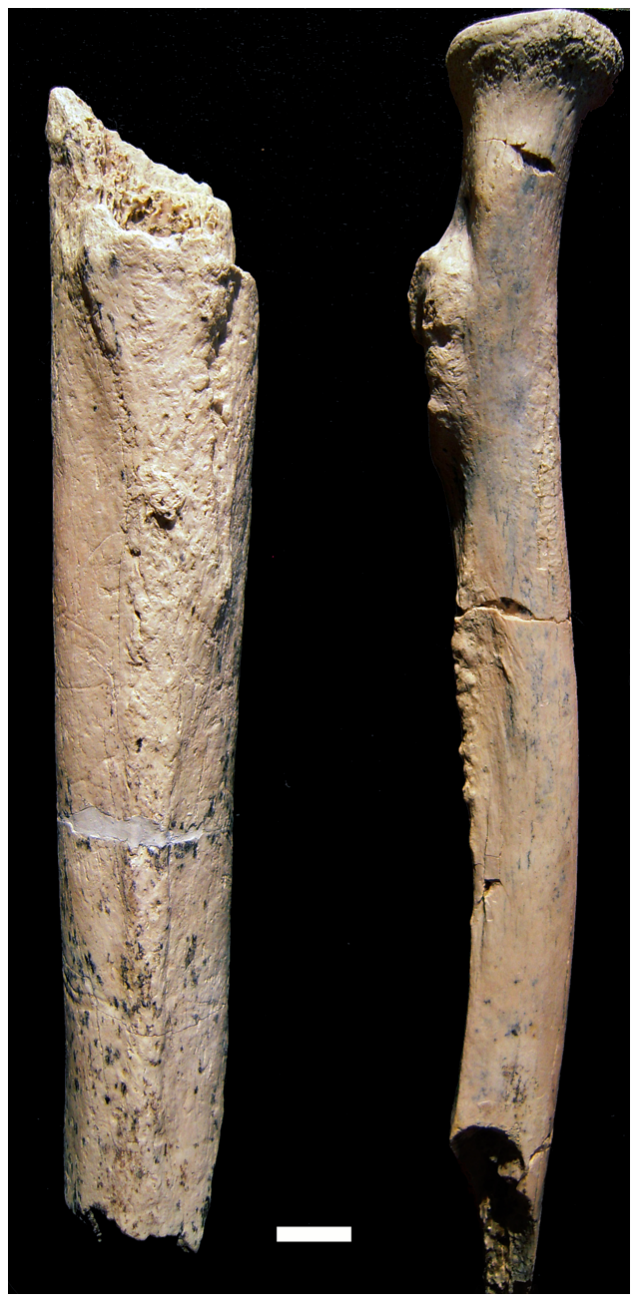
The right femur (OH 80-12; left side of image) and right radius (OH 80-11; right side of image) of the OH 80 hominin from Level 4 at the BK site. Both fossils are shown in posterior view; superior is at the top of the image; the bar scale = 1 cm.

**Figure 3:**
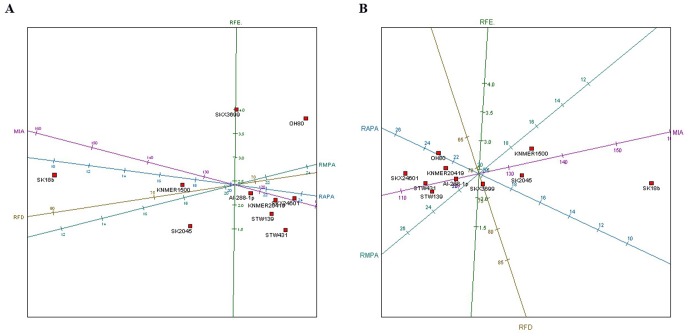
PCA showing the OH80 radius compared to other australopithecine and Homo fossils. A, confirmatory PCA of the variables analyzed for several fossil hominin proximal radial specimens (excluding the variable AP ratio, since predictive diagrams suggested that it was not diagnostic and that it had three times more predictive error margin than the variable with the smallest error and 50% more error than the other variables). The PCA yielded a two-component solution, which accounted for 91.9% of the sample variance. Dimension 1 accounted for 70% of the sample variance; dimension 2 explained the remaining 21.9% of variance. The variables showing the highest scores in factor 1 were MIA (.51), RAPA (−.50), RMPA (−.48) and RFD (−.47). In the second factor, RFE yielded the highest score (.97). B, A similar distribution of the sample can be observed in a MDS analysis, which reached a solution after 77 iterations, consisting of a two-dimensional solution explaining 89.2% of sample variance. The first dimension (71% of inertia) showed the same variables selected as in the PCA but with different scores: MIA (.89), RAPA (.28), RMPA (−.23), RFD(.24). The second dimension is defined by RFD (−.73). Abbreviations: medial proximal-distal intersecting angle (MIA); relative fovea diameter (RFD); relative fovea eccentricity (RFE); relative anterior proximal articular surface width (RAPA); relative medial proximal articular surface width (RMPA) [25]. Horizontal axes display the first dimension and vertical axes show the second dimension of data. Fossils (catalog number, species): KNM-ER 20419, *Australopithecus anamensis*; AL-288-1p, *Australopithecus afarensis*; StW 431, *Australopithecus africanus*; StW 139, *Australopithecus africanus*; SKX 3699, *Paranthropus robustus*; SKX 24601, *Paranthropus robustus*; KNM-ER 1500, *Paranthropus boisei*; SK18b; *Homo erectus*; SK 2045, (?) *Homo erectus*.

OH 80-11 is a partial right radius, 194 mm in maximum length, with a head, proximal metaphysis and partial diaphysis (Figure 2). The specimen was recovered from a hard concretion matrix. After removal of the encasing matrix, several dry cracks are observable on the bone's cortex; otherwise, its overall surface preservation is fairly good. It lacks subaerial weathering (stage = 0). Although the specimen does not preserve tooth marks, carnivore(s) may have removed its distal end, as its shaft terminates in a green bone fracture. Compared to anatomically relevant *Australopithecus* (KNM-ER 20419, AL-288-1p, StW 431, StW 139) and presumed *Paranthropus* (KNM-ER 1500, SKX 3699, SK 24601) radius specimens, OH 80-11 is extremely robust, absolutely larger than any other known hominin radius fossil from the Pliocene or early Pleistocene (Figures S3 and S4 in Appendix S1). Its ML head diameter is 25.3 mm; its AP head diameter is 26.3 mm. Its articular fovea occupies an eccentric position on the head, with a large beveled margin along the anterior and medial rim of the articular surface, between its proximal and distal portions. This morphology indicates functional stability in the humeroradial and proximal radioulnar joints, as is typical of extant African apes and Asian hylobatids, as well as of all inferred non-*Homo* hominin radii currently known [25] (Figures 3 and S4 in Appendix S1). Comparatively, the Swartkrans proximal radius specimen SK 18b, inferred to represent early Pleistocene *H. erectus* [26], [27], shows a straighter angle between the proximal and distal portions of its articular surface and lacks a marked bevel on its anterior and medial sides [24], [25], as seen in OH 80-11. In addition, the articular fovea of SK 18b is more centrally positioned, as in modern *Homo*, and in contrast with *Australopithecus* and presumed *Paranthropus* radii [25], [28], including OH 80-11.

The neck of OH 80-11 is long (24.4 mm from the distal articular surface to the superior margin of the bicipital tuberosity), as is typical of several inferred non-*Homo* hominin radii [28], [29]. The neck is also relatively constricted mediolaterally (14.8 mm), but quite robust anteroposteriorly (19.1 mm). The dimensions of the neck, as well as those of the bicipital tuberosity (maximum superoinferior length = 33.9 mm; maximum transverse width = 19.0 mm), are absolutely and relatively larger than those documented for any other Pliocene and Pleistocene hominin radius specimen [24], [25], [28], [29]. The overall massiveness of OH 80-11, and specifically that of its bicipital tuberosity, suggests that the individual from which the specimen derived possessed large biceps brachii muscles and the ability for application of great strength in forearm flexion. In contrast to the more anteriorly positioned bicipital tuberosities of definitive *Homo* radii, OH 80-11 shows a more medially placed tuberosity. However, the longitudinal axis of OH 80-11's bicipital tuberosity does not intersect the axis of its interosseous crest, as is common in nonhuman apes and in some Pliocene and Pleistocene hominin radial specimens [30], [31]. The longitudinal axis of the interosseous crest of OH 80-11 is posterior to its bicipital tuberosity. Thus, if we are correct in our assignment of OH 80-11 to *P. boisei* (see below), then, necessarily, the posterior position of this axis in *H. sapiens* is not an autapomorphy.

The interosseous crest of OH 80-11 is strongly marked and curved medially, as in other early hominin radius specimens inferred to derive from taxa other than *Homo* [24], [28]. Its inferior trajectory also curves posteriorly, possibly positioning the crest axis as well as the tuberosity anteriorly to the ML axis of the articular surface of the distal epiphysis, as in modern humans and in contrast with apes and various species of Pleistocene *Homo* [32]. The proximal portion of the crest shows a medially projecting, superoinferior line of enthesophytic growth. A prominent posterior oblique line opposite the interosseous crest suggests a well-developed pronator teres muscle.

On balance (and continuing to recognize the tenuousness of the taxonomic status of some other early hominin comparative radius specimens), most of the characteristics of OH 80-11 described above suggest that it derives from a non-*Homo* species. Further, because *Australopithecus* is not known from OH 80-11's temporogeographic context, its most parsimonious taxonomic assignment is to *P. boisei* (Figure 3). The only differences between OH 80-11 and other inferred *Paranthropus* and *Australopithecus* radius specimens are simply dimensional. The configuration of the anteromedial rim of the head's articular surface explains most of the differences between OH 80-11 and other Pliocene and Pleistocene hominin proximal radius specimens (Figures 2 and 3). Small, posterolaterally displaced articular foveae are a plesiomorphic feature in African apes, in inferred non-*Homo* fossil hominins [25] and in OH 80-11. In contrast, *H. sapiens* and inferred early *Homo*, such as SK 18b, possess relatively large and centrally-positioned articular foveae with very obtuse angles at the anteromedial section of the articular surface (Figure S3 in Appendix S1). Radial head diameter has been used to derive body weight estimates [33], but we are unconvinced by the efficacy of this approach for bipedal primates and thus do not present relevant data here.

### Femur

OH 80-12 is a diaphyseal segment of a right femur (Figure 2). The specimen was recovered in three refitting pieces that join to form a maximum length of 167.0 mm. As with the other OH 80 postcranial specimens, OH 80-12 is not weathered (stage 0), but is covered by a few deposits of manganese dioxide. The reconstructed specimen retains its complete diaphyseal circumference and is truncated by two green bone fracture edges. As distinctive carnivore tooth marks scar the cortex of OH 80-12, we infer that the fracture edges were also created by carnivores. This inference is supported by crenulation, a common taphonomic result of carnivore chewing, on the specimen's proximal fracture edge. Proximally, the specimen ends just inferior to the missing lesser trochanter. The proximodorsal surface of the specimen preserves the gluteal, pectineal and spiral lines (the first two strongly marked and the latter less defined). These lines converge into a prominent *linea aspera* distally, which carries on to the specimen's distal termination, superior to the popliteal surface. The diaphyseal cortex of OH 80-12 is extremely thick (range = 8.3–11.0 mm mid-diaphysis), and the specimen displays a very constricted medullary cavity. Periosteally, OH 80-12 shows some flattening at its proximal end (ML = 30.5 mm; AP = 23.0 mm), with a platymeric index of 0.75.

Day [34] and Walker and Leakey [35] suggest that a thick, platymeric femur shaft is a taxonomically diagnostic feature of early *Homo*. However, given that hominin femur specimens have been assigned to *Paranthropus* based mainly on the morphology of their proximal epiphyses and metaphyses (i.e., it is argued that *Paranthropus* [and *Australopithecus*] femora have small heads and long, anteroposteriorly constricted necks) [7], [34], and that no complete or nearly complete femur fossils (i.e., specimens with sizeable lengths of their diaphysis preserved) were previously associated exclusively with *P. boisei* craniodental remains [33], the femur shaft morphology of *Paranthropus* is actually poorly known. Further, some *H. habilis* femur specimens (e.g., OH 62) have morphology similar to that described for *Paranthropus* [7], [33], [36].

Swartkrans fossils SK 82 and SK 97, two femoral specimens preserving segments of proximal diaphyses and that are attributed to *P. robustus*, show impressive cortical thickness (comprising 77% to 85% of their respective total sections) [37]. SK 82 and SK 97 are also fairly platymeric, with respective platymeric indices of 0.77 and 0.80. Assuming that SK 82 and SK 97 are, in fact, *P. robustus* fossils, then these observations suggest that neither platymeria nor diaphysis cortical thickness is a useful trait for separating early *Homo* from (at least some) non-*Homo* hominin femora [37]. OH 80-12 shares with SK 82 and SK 97 a diaphysis morphology that places the group apart from *H. erectus* [38] (Table 2 and Figures S5, S6, S7 in Appendix S1). The distribution of cortical bone inferior to the lesser trochanter of OH 80-12 represents 85.7% of its shaft section, far from the mean and outside the range for six early *H. erectus* infratrochanteric femoral sections measured at 80% of femoral length [37]. Values for OH 80-12's ML bending rigidity and polar second moment of area are also more similar to those of SK 82 and SK 97 than to those of *H. erectus*. Compared to the only adult early *H. erectus* femur specimen for which measurements at 50% shaft section are available [37], OH 80-12 has a thicker mid-diaphysis cortical section (>90% of total section area), maintaining the inter-taxonomic differences in ML bending rigidity and polar second moment of area as observed with comparisons of infratrochanteric sections (Table 3). In sum, OH 80-12 possesses the thickest femoral diaphysis of all currently known hominins from contexts >1.0 Ma.

**Table 2:** Section properties of proximal femora (at 80% length[1 cm inferior to the lesser trochanter]) of two *Paranthropus robustus* specimens [37] and OH80.

Specimen	CA	MA	TA	%CA	I_X_	I_Y_	J
SK 82	490	87	577	84.9	21.637	31.261	52.899
SK 97	457	135	593	77.1	21.228	33.779	55.007
OH 80-12*	524	87	611	85.7	26.234	32.458	58.691

CA, cortical area; MA, medullary area; TA, total periosteal area; %CA, [(CA/TA) · 100); Ix, second moment of area about X (ML) axis (AP bending rigidity); Iy, second moment of area about y (AP) axis (ML bending rigidity); J, polar second moment of area. Areas in mm^2^, second moments of area in mm^4^.

* Measurements from the proximal shaft were obtained near the top of the preserved section, slightly at the same undertrochanteric section as suggested by Ruff et al. [37].

**Table 3:** Cross-sectional properties of femoral 50% sections of OH80 and KNM-ER 1808 (*Homo erectus*).

Specimen	CA	MA	TA	%CA	I_X_	I_Y_	J
OH 80-12*	492	35	526	93.5	21.763	22.161	43.924
KNMER 1808	478	73	551	86.8	20.813	27.251	48.064

CA, cortical area; MA, medullary area; TA, total periosteal area; %CA, [(CA/TA) · 100); Ix, second moment of area about X (ML) axis (AP bending rigidity); Iy, second moment of area about y (AP) axis (ML bending rigidity); J, polar second moment of area. Areas in mm^2^, second moments of area in mm^4^.

* Measurements taken directly on the proximal break of the shaft at the mid-section.

### Tibia

OH 80-13, a hominin tibia midshaft fragment (retaining <50% of its original circumference), is described in the Appendix S1(Figure S8). Based its close spatial association, comparable anatomical ruggedness and similar state of preservation to the other OH 80 postcranial fossils, described above, we hypothesize that OH 80-13 derived from the same hominin skeleton as did the rest of those fossils.

## Conclusions

Wood and Constantino [7] provide a sustained and compelling deconstruction of previous claims of postcranial fossils of *P. boisei*. The demonstrated lack of confidently assigned *P. boisei* postcranials severely limited our understanding of this extinct taxon's biology. Here, we argue that OH 80, the partial hominin skeleton from Level 4, BK, Olduvai Gorge, is very likely that of *P. boisei*. We base these propositions—i.e., that the collective remains sample one adult individual and that that individual is represents the species *P. boisei*—on the principle of taphonomic parsimony. All hominin specimens were recovered *in situ* from the same depositional stratum, the same paleosurface and in close horizontal proximity (Figure S9 in Appendix S1). The remains display an identical state of preservation and the postcranial fossils, which are all inferred to have been modified by carnivores, derive from a larger fossil assemblage and larger site in which carnivore modification of fossils is overall not very conspicuous [10]. Allocation to *P. boisei* of the best preserved dental remains is based on long-tested and well-accepted diagnostic morphological criteria (discussed above in the descriptions). In addition to the close vertical and horizontal spatial linking of these teeth with the postcranial fossils, the postcranials, themselves, show many morphological characteristics that ally them most closely to homologous fossils inferred to derive from non-*Homo* taxa, and that distinguish them from known early *Homo* homologs.

If our taxonomic hypothesis is correct, then the analysis of OH 80 provides new insights to *P. boisei* biology as follows. First, using a minimum dimension of the linea aspera (83.60 mm) as defined in [39], an estimate of 400 mm for the complete OH 80 femur was obtained using the regression formulae from [39]. Based on this reconstructed femoral length, the hominin's minimum stature is estimated to have been 156±3.91 cm. We stress, however, that this estimate was derived from regression formulae based on the dimensions of modern human *linea asperae* [39], and that modern human body proportions are probably not adequate proxies for those of *Paranthropus*. We also recognize that because the OH 80 femur lacks epiphyses (among the best postcranial proxies of body mass), its relevance for further elucidating *P. boisei* body size dimorphism (and its sociobehavioral implications) is limited. With that caveat, application of McHenry's [33] femoral shaft module regressions (using least square formulae) to OH 80-12 yielded the value 701.5, which corresponds to a 50.0 kg human (or 40.0 kg after application of the 0.74 correction of estimate, recommended by McHenry) or to a 61.7 kg non-human hominoid—significantly heavier than estimates for KNM-ER 1500, a presumed female *P. boisei*. This tenuous contrast between the larger, presumed male (OH 80) and smaller, presumed female KNM-ER 1500 postcranium [8], agrees with other postcranial- and skull-based inferences that *P. boisei* was substantially sexually dimorphic in body size [40]–[44].

Second, comparative analyses of OH 80-12 also demonstrate dimensional overlap in the femora of *P. boisei* and *H. erectus* [45]. In addition, the OH 80-12 femur shares with *H. erectus* femora (e.g., OH 28; [46]) the following features: significant cortical thickness; a platymeric proximal diaphysis; similar arrangement of the gluteal, pectineal and spiral lines. While these findings urge caution for taxonomic allocation of isolated hominin postcranial specimens in geographies where *P. boisei* and *H. erectus* overlapped temporally, other distinguishing features of the two species' femora are apparent. For instance, the spiral line is significantly more marked in OH 80-12 than in known *H. erectus* femora [46]. Further, the gluteal tuberosity of OH 80-12 is more medially oriented than are those of most *H. erectus* femora, which frequently possess well-developed hypertrochanteric fossae and lateral expansions of their gluteal tuberosities, forming convexities that contrast with the concave outlines of their femoral mid-diaphyses [46]. In contrast, the lateral outline of the mid-diaphysis of OH80-12 is straighter (Figure S6 in Appendix S1). These morphological differences probably reflect variance in the biomechanics of the lower limbs of *P. boisei* and *H. erectus*.

**Figure 4:**
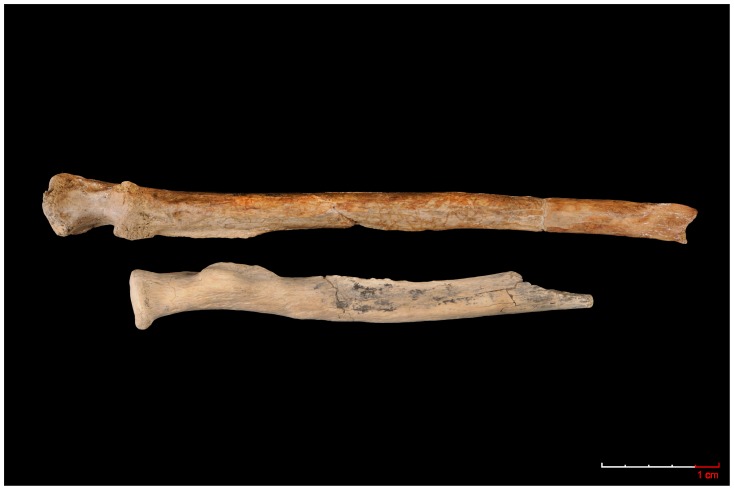
Ulna (OH36) found in upper Bed II and attributed to Paranthropus (upper) compared to the OH80-11 radius (lower) (Photo: Mario Torquemada).

Third, morphological analyses of the OH 80-11 radius support the claim that *P. boisei* “had relatively large and powerfully built forelimbs” (ref. [33] p. 427), larger than expected based on lower limb size [41]. The OH 80-11 radius is the most robust forearm bone currently known in the Pliocene and early Pleistocene hominin fossil record. The OH 36 ulna, which was previously tentatively attributed to *P. boisei*, is also powerfully built, with extensive buttressing posterior to its trochlear notch, substantial height and width (beyond the articular surface) of its olecranon, marked curvature of its shaft and a rugged brachial tuberosity [47] (Figure 4).

In addition, the morphology of OH 80-11's proximal articular surface, with lateral eccentricity of its articular fovea, is similar to those of climbing pongids and hylobatids [25]. The morphology of hand bones attributed to South African *P. robustus* is not indicative of regular suspensory activities [29]. Thus, if *P. robustus* and *P. boisei* are monophyletic (a proposition that we do not necessarily accept), then possible regular climbing *P. boisei* climbing is a potential explanation for presumed plesiomorphic, but actually apomorphic, climbing features of the OH 80-11 radius.

No hand bones are securely attributed to *P. boisei*, but the radial morphology of OH 80 is not incompatible with that hominin having possessed similar grasping capabilities as did/does *Homo*, employed for both power and precision grips that are associated with intricate manual manipulations. For context, Susman [29] argued, based analyses of hominin hand bones from Swartkrans, that *P. robustus* was also a tool-maker and -user [29]. (Some of the current authors believe, though, that *H. erectus* was the most likely manufacturer, user and depositor of stone tools at BK.)

In sum, the OH 80 *P. boisei* teeth, from Level 4 at the BK site, establish a new last appearance datum of 1.338±0.024 Ma for that species. Based on taphonomic parsimony, three long limb bone fossils are derived from the same *P. boisei* skeleton as are the OH 80 teeth. These skeletal remains indicate that *P. boisei*—a craniodentally highly specialized species, cofamilial and sympatric with the genus *Homo*—had a robust postcranium, and probably combined terrestrial bipedalism with at least occasional bouts of arboreality.

## Methods

### Ethics statement

The analysis was carried out on the following fossils: dentition (from OH 80-1 to OH 80-9), humerus (OH 80-10), radius (OH 80-11), femur (OH 80-12) and tibia (OH 80-13). These fossils are stored at the National Museum of Tanzania at Dar es Salaam. All necessary permits were obtained for the described study, which complied with all relevant regulations. Research was conducted with permits from the Tanzania Commission for Science and Technology (COSTECH) (n° 2013-181-ER-2006-115) and The Antiquities Department of the Ministry of Natural Resources and Tourism (Dar es Salaam, Tanzania) (n° 03/2006-2013).

### Methodology

Each dental specimen was examined using a low-power (10x–50x) binocular microscope and, when possible, measurements were taken with a PaleoTech Concepts™ Hillson-Fitzgerald digital dental caliper. Standard gross tooth crown measurements were taken to the nearest tenth of a millimeter (e.g., [18], [48], [49]). Taxonomic determinations of teeth were based on various features used previously to differentiate *Australopithecus*, *Paranthropus* and early *Homo* dentition, including, but not limited to: number of cusps; relative cusp proportions and occlusal wear pattern (e.g., [18], [48]–[55]). The postcranial fossils were examined macro- and microscopically and measurements were taken on them using a Mitutoyo™ digital caliper.

Several variables related to radius head morphology were selected for the study of OH 80-11, the radius specimen, including: anteroposterior (AP) ratio; medial proximodistal intersecting angle (MIA); relative articular fovea diameter (RFD); relative articular fovea eccentricity (RFE); relative anteroproximal articular surface width (RAPA); relative medioproximal articular surface width (RMPA). All variables but AP ratio were proposed and defined by Patel [25]. Metric measurements of the head and neck followed Senut [56].

A principal component analysis (PCA) was conducted to document relationships among the components of the sample. PCA produce factors that result from the reduction of dimensionality caused by multiple variables. PCA are used in exploratory and confirmatory ways. Exploratory PCA aims to improve prediction and variance accountability by detecting those variables that do not contribute significantly to sample variance. Confirmatory PCA uses selected variables to maximize sample variability and sample component relationship. Given the use of continuous numerical variables, the heterogeneity of these values may bias PCA solutions by overemphasizing the weight of variables displaying high numerical values. For this reason, all variables were centered and scaled prior to their statistical analysis.

As opposed to dimension reduction by orthogonal projection as performed in PCA, in multidimensional scaling (MDS) the points are chosen so that stress (the sum of the squared differences between the inter-sample disparities and the inter-point distances) is minimized [57]. The MDS option we selected for our analyses is the identity transformation, which consists of taking the inter-sample disparities to be the inter-sample dissimilarities themselves. This metric MDS approach uses a Pythagorean metric analysis of inter-point distances, which includes an iterative majorization algorithm to find the MDS solution [57]. This algorithm was considered to have converged as soon as the relative decrease in stress was less than 10^−6^. The algorithm was also stopped once greater than 5,000 iterations were performed. In MDS, points are related in a low-dimensional Euclidean space [57], with data spatially projected by regression methods admitting non-linearity. The use of MDS in the present study, thus, complements the PCA test.

A canonical variate analysis (CVA) was also used (see Appendix S1). CVA focuses on data grouped into K classes by transforming original variables into canonical variables defined by square distances between the means of the groups obtained by Mahalanobis's D^2^. This is scale invariant. CVA produces a higher degree of separation between the group means than either PCA or MDS [58]. In CVA, biplot axes are determined by the group means.

Analyses were performed in R. Graphic display of PCA and MDS tests were carried out with biplots using the R library “BiplotGUI”. Bivariate display of AP and ML measurements was produced with the “ggplot2” R library. Graphic display of multiple correspondence analysis biplot with categorical variables for femora was conducted with the UBbipl R library [59].

For the analysis of the OH 80-12 femur specimen, measurements were taken directly on the proximal shaft, which exhibits a section slightly inferior to the missing lesser trochanter, and on the mid-diaphysis thanks to the natural breaks exposing these sections. In addition, after restoration of the bone, CT scanned sections were taken at the mid-diaphysis section. Average estimates after six repeated measurements were taken in each case, first with digital calipers and then with image software (IQ View Pro 2.6.0). Geometric section properties were obtained from biplanar external and cortical breadths using an Eccentric Ellipse Model [60]. Measurements were processed using the EEM_Macro (http://www.hopkinsmedicine.org/fae/CBR.htm).

## Supporting Information

Appendix S1 **Supporting information.** Figure S1. CT-scan image of AP section of the hominin distal humerus diaphysis (OH 80-10). Although the strong density limits the quality of the image, it clearly shows that the specimen's cortex is thick and its medullary cavity is narrow and full of trabecular bone. Figure S2. Differences between distal humeri attributed to *Homo* and to *Paranthropus* from Swartkrans (1) (upper half) and OH 80-10 (lower half). Note the elongated section distally in OH 80-10, above the biepicondylar line, which extends to the red line. Figure S3. Canonical Variable Analysis (CVA) of australopithecine (Australopithecus+Paranthopus) and Homo proximal radial specimens. CVA increases the inter-group sample differences compared to PCA and MDS. A, CVA including SK2045, which is tentatively identified as Homo sp by morphology, in contrast with SK8b (which is associated with dental remains and, thus, more securely identified). However, SK2045 is spatially placed closer to other australopithecine specimens (see main text, Figure 2), indicating that the morphology of its articular surface is ambiguous. B, CVA of the same hominin radial sample removing SK2045. Differences between Homo and australopithecines are increased. OH80-11 seems thus even closer to the latter. The average amount of predictive error in each variable is reduced from 14% in A to 10% in B. Figure S4. Dimensions (AP = anterior-posterior; ML = medial-lateral) of the proximal articular head of hominin radii of different hominins. Measurements for modern humans are from Senut [3] and for Neanderthal and Atapuerca specimens are from Carretero [4]. Abbreviations: Krp, Krapina; Ferr, Ferrasie; SKX, Swartkrans; STW, Sterkfontein; AT, Atapuerca. Bars for modern humans and Neanderthals show maxima and minima. Figure S5. Multiple correspondence analysis (MCA) of OH 80-12 and the femora of *Homo erectus*. Abbreviations: HTF = hyper-trochanteric fossa; LEGT+lateral extension of the gluteal tuberosity; LAP = *linea aspera* position. *Homo erectus* data from [5]. Figure S6. Comparison of OH 80-12 (right) and three representative femora of *Homo erectus* [5], illustrating different expressions of taxonomically diagnostic criteria. Note OH 80-12's lack of a hyper-trochanteric fossa and its more medially placed *linea aspera*, which is also more robust than those of *H. erectus*. Figure S7. CT-scan image of the AP section of OH 80-12 (left) and of diaphysis section (center, right), illustrating the robust thickness of the diaphysis and thickness distribution (second moment of area) according to orientation: A = anterior; P = posterior; L = lateral; M = medial. The femur section is oriented distally (upper) to proximally (lower). The diaphysis section (center,right) is taken at the mid-diaphysis, coinciding with the natural fracture as seen on the left scan. A diaphysis section of a the KNM- ER 1808 *Homo erectus* femur is overlaid (lower right) to show differences. Note the proportionally smaller medullary cavity and more thicker cortex of OH 80-12. Figure S8. OH 80-13 (tibial shaft) compared to a modern human tibia. Image by E. Organista and JL Heaton. Figure S9. Stratigraphic sequence of Upper Bed II, Olduvai Gorge, and location of the BK site (right), with isometric reconstruction showing level 4 and a map with the distribution of materials and location of each of the excavated hominin fossils. Figure S10. Map of Olduvai Gorge showing the localities where the tuff IID samples were collected. Figure S11. Photographs of Tuff IID in each of the localities and stratigraphic position of the tuff at each of them. Figure S11. Photographs of Tuff IID in each of the localities and stratigraphic position of the tuff at each of them. Figure S12. Ar/Ar results for the BK sample. Figure S13. Ar/Ar results for the RHC sample. Figure S14. Ar/Ar results for the MCK sample. Figure S15. Ar/Ar results for the JK sample. Figure S16. Compositions of mineral phases in the Olduvai tuff samples. (a) Ab-rich anorthoclase and plagioclase feldspar compositions. (b) Augite and sodic augite compositions. (c) Spinel and rhombohedral (Ti-rich) Fe-Ti oxide compositions. (d) Hornblende compositions. Loading scores (Table S1). Loading scores (Table S2). Loading scores (Table S3). Loading scores (Table S4). Loading scores (Table S5). Table S6. Ar/Ar results are summarised in the table below and ages are calculated using two different sets of standard ages and decay constants. Table S7. Mineralogy and composition of some phases in the Olduvai Gorge (Tanzania) samples.
(PDF)
