# Supplementary material for: First Partial Skeleton of a 1.34-Million-Year-Old Paranthropus boisei from Bed II, Olduvai Gorge, Tanzania
Source: PLoS One. 2013 Dec 5;8(12):e80347. doi: 10.1371/journal.pone.0080347 (PMC3855051; doi:10.1371/journal.pone.0080347)
Supplement: Appendix S1 — Supporting information. Figure S1. CT-scan image of AP section of the hominin distal humerus diaphysis (OH 80-10). Although the strong density limits the quality of the image, it clearly shows that the specimen's cortex is thick and its medullary cavity is narrow and full of trabecular bone. Figure S2. Differences between distal humeri attributed to Homo and to Paranthropus from Swartkrans (1) (upper half) and OH 80-10 (lower half). Note the elongated section distally in OH 80-10, above the biepicondylar line, which extends to the red line. Figure S3. Canonical Variable Analysis (CVA) of australopithecine (Australopithecus+Paranthopus) and Homo proximal radial specimens. CVA increases the inter-group sample differences compared to PCA and MDS. A, CVA including SK2045, which is tentatively identified as Homo sp by morphology, in contrast with SK8b (which is associated with dental remains and, thus, more securely identified). However, SK2045 is spatially placed closer to other australopithecine specimens (see main text, Figure 2), indicating that the morphology of its articular surface is ambiguous. B, CVA of the same hominin radial sample removing SK2045. Differences between Homo and australopithecines are increased. OH80-11 seems thus even closer to the latter. The average amount of predictive error in each variable is reduced from 14% in A to 10% in B. Figure S4. Dimensions (AP = anterior-posterior; ML = medial-lateral) of the proximal articular head of hominin radii of different hominins. Measurements for modern humans are from Senut [3] and for Neanderthal and Atapuerca specimens are from Carretero [4]. Abbreviations: Krp, Krapina; Ferr, Ferrasie; SKX, Swartkrans; STW, Sterkfontein; AT, Atapuerca. Bars for modern humans and Neanderthals show maxima and minima. Figure S5. Multiple correspondence analysis (MCA) of OH 80-12 and the femora of Homo erectus. Abbreviations: HTF = hyper-trochanteric fossa; LEGT+lateral extension of the gluteal tuberosity; LAP = linea asp [file pone.0080347.s001.pdf]

SUPPLEMENTARY INFORMATION

INDEX

GRAPHIC DOCUMENTATION OF THE STUDY OF THE OH-80 HOMININ POSTCRANIAL FOSSILS........................................................................................................................ p. 2
THE GEOLOGICAL CONTEXT OF BK ......................................................................p. 13
TUFF IID ........................................................................................................................p. 16
Ar/Ar METHODOLOGY AND RESULTS.....................................................................p. 19
TEPHROCHRONOLOGY OF TUFF IID .......................................................................p. 25
REFERENCES...............................................................................................................p. 27

**GRAPHIC DOCUMENTATION OF THE STUDY OF THE OH 80 HOMININ POSTCRANIAL FOSSILS**

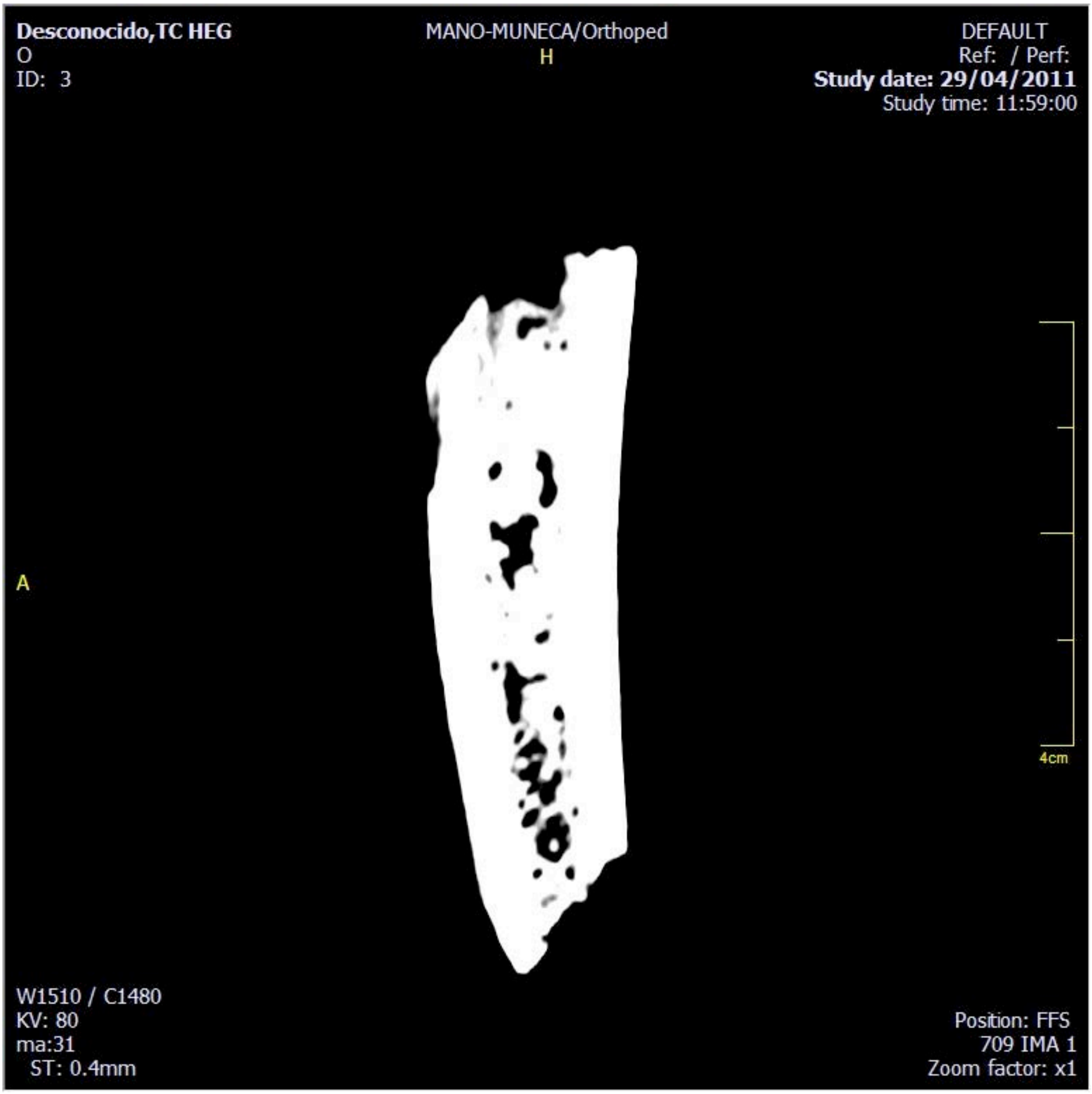


Figure S1. CT-scan image of AP section of the hominin distal humerus diaphysis (OH 80-10). Although the strong density limits the quality of the image, it clearly shows that the specimen's cortex is thick and its medullary cavity is narrow and full of trabecular bone.

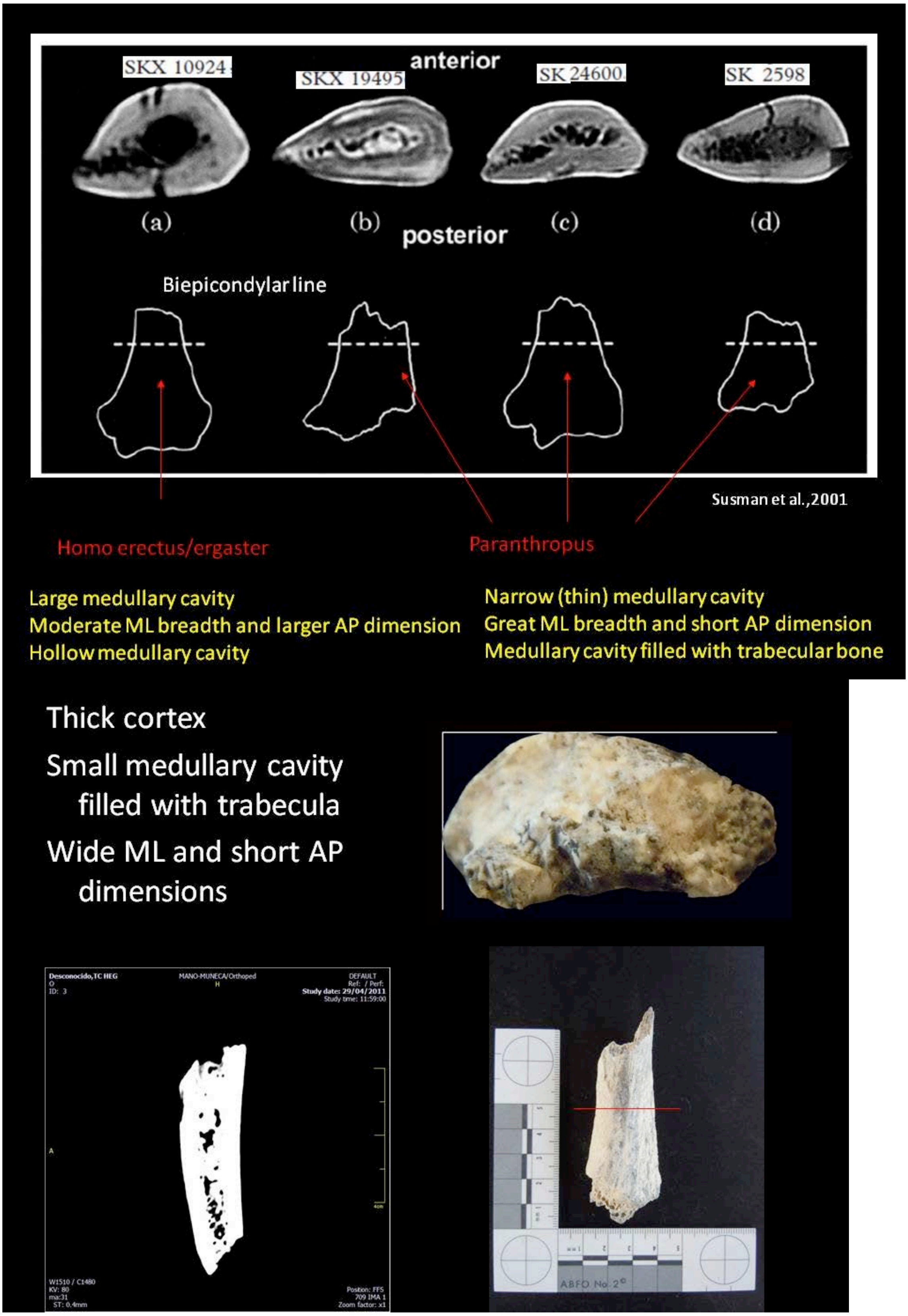


Figure S2. Differences between distal humeri attributed to *Homo* and to *Paranthropus* from Swartkrans (1) (upper half) and OH 80-10 (lower half). Note the elongated section distally in OH 80-10, above the biepicondylar line, which extends to the red line.

**Statistical analysis of the radius (OH80-11)**

*Exploratory PCA*

A total of 77.9% of the variation in the samples is accounted for by the first two principal components. Dimension 1 accounted for most of the variance (61%). The variables showing the highest scores in Dimension 1 were MIA (.51), RAPA (-.50) and RMPA (-.48). The highest score for variable 2 was observed in RFE (.88).

Predictive diagrams suggested that AP ratio was the least diagnostic variable. This was supported by the relative absolute error, which showed that this variable had three times more predictive error margin than the variable with the smallest error (MIA) and 50% more error than the other variables.

Loading scores (Table S1):

| | Dimension 1 | Dimension 2 |
|---|---|---|
| AP.MD_index | 0.14570497 | 0.43645798 |
| RFD | 0.47069667 | -0.02772051 |
| RFE | -0.00781116 | 0.88359833 |
| RMPA | -0.48673026 | 0.14931004 |
| RAPA | -0.50856012 | 0.01689980 |
| MIA | 0.51148210 | 0.07355891 |

*Confirmatory PCA and MDS (main Text, Figure 3A and B)*

A second PCA, excluding the "AP ratio" variable yielded a two-factor solution in which the first two dimensions accounted for 91.9% of the sample variance. Factor 1 accounted for 70% of the sample variance. The variables that showed the highest scores in the first exploratory PCA (MIA, RAPA, RMPA) showed similar scores in the second confirmatory PCA. In the second dimension, RFE yielded a higher score than in the first PCA (.97).

Loading scores (Table S2):

| | Dimension 1 | Dimension 2 |
|---|---|---|
| RFD | -0.47038547 | -0.07204365 |
| RFE | 0.01138304 | 0.97789326 |
| RMPA | 0.49208566 | 0.12394264 |
| RAPA | 0.51794861 | -0.06900804 |
| MIA | -0.51786956 | 0.13568565 |

This result shows that the configuration anterior-medial rim of the articular surface (RAPA, RMPA) and the medial angle of the proximal and distal articular surface (MIA) explains most of the differences among the specimens analyzed. Given the structural relationship of these variables with the eccentricity of the fovea (RFE), it is not surprising that the latter accounts for most of the second factor. Thus, large and centered foveae with very obtuse angles in the medial-anterior section of the articular surface are typical of *Homo* radius specimens (e.g., SK18b) [1], smaller and more posterior-laterally displaced foveae are typical of non-*Homo* radius specimens. This is a plesiomorphic feature also found in African apes [2].

A similar distribution of the sample was observed in a MDS analysis, which reached a solution after 77 iterations, consisting of a first dimension with the same variables selected as in the PCA (MIA, RMPA, RAPA) with the highest scores, and a second dimension defined by RFE.

Loading scores (Table S3):

| | Dimension 1 | Dimension 2 |
|---|---|---|
| RFD | 0.243449509 | -0.73989912 |
| RFE | 0.005078331 | 0.08742016 |
| RMPA | -0.232438149 | -0.19134563 |
| RAPA | -0.284147280 | 0.13346112 |
| MIA | 0.891612901 | 0.19605597 |

Canonical variable analysis (Figure S3, below)

A CVA was conducted comparing the Australopithecus and Paranthropus radius sample with SK2045, tentatively identified as Homo sp by morphology [1] (in contrast to SK8b, identified as Homo because of the association with dental remains, which is also included in the comparative sample). The two-dimension solution explained 90.7% of variance (first dimension accounted for 69.9% of sample variance). The first CVA produced the following two-dimension solution:

Loading scores (Table S4):

| | Dimension 1 | Dimension 2 |
|---|---|---|
| RFD | -4.5940328 | -1.3201891 |
| RFE | 0.1739488 | 0.0530875 |
| RMPA | 3.3897718 | 1.7210520 |
| RAPA | 4.4111635 | 1.4143606 |
| MIA | -14.7633482 | 0.3416385 |

The resulting CVA produced some distortion on data because of the ambiguity provided in the identification and metric attributes of SK2045. For this reason, a new CVA excluding SK2045 was conducted with the following results.

Loading scores (Table S5):

| | Dimension 1 | Dimension 2 |
|---|---|---|
| RFD | -3.08028257 | -1.872128 |
| RFE | -0.07361682 | -0.554642 |
| RMPA | 2.25887388 | -4.985230 |
| RAPA | 2.82456100 | -3.313567 |
| MIA | -9.53144040 | 10.350273 |

This result supports the attribution of OH 80-11 to the group composed of *Australopithecus* and *Paranthropus* radii (Figure S3).

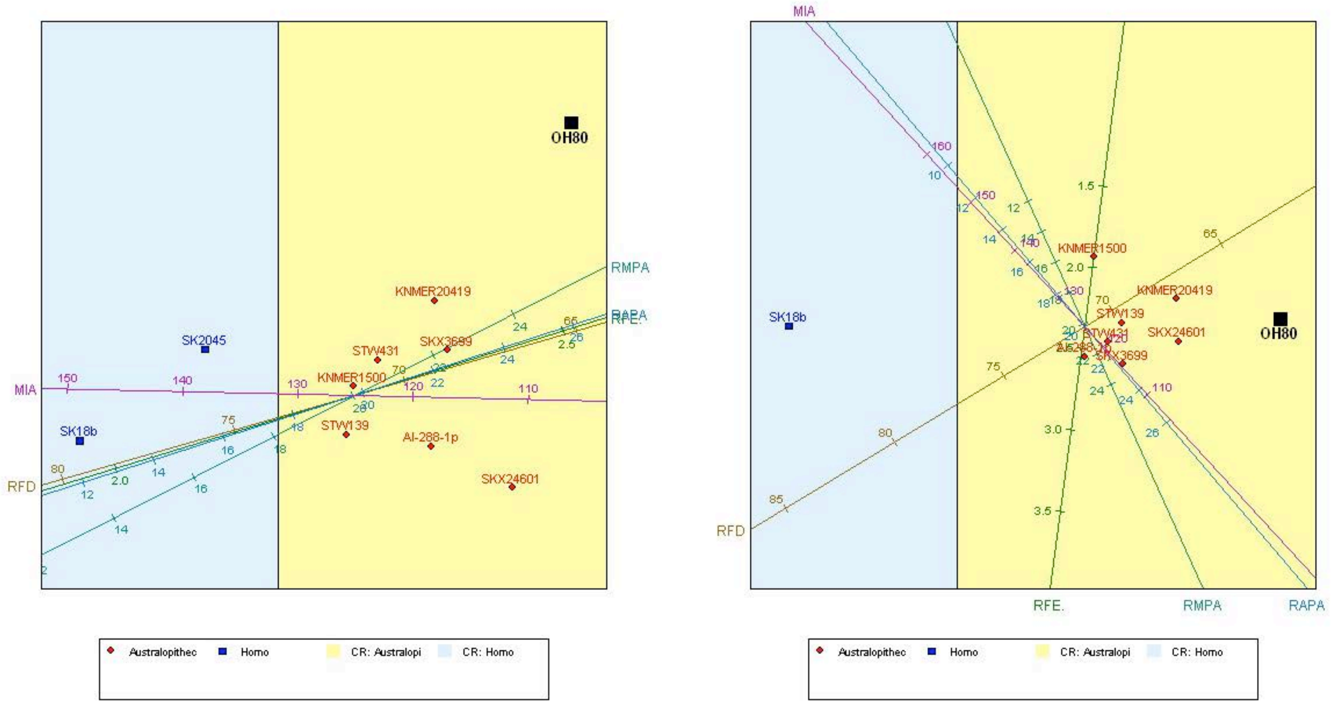


Figure S3. Canonical Variable Analysis (CVA) of australopithecine (Australopithecus+Paranthopus) and Homo proximal radial specimens. CVA increases the inter-group sample differences compared to PCA and MDS. A, CVA including SK2045, which is tentatively identified as Homo sp by morphology, in contrast with SK8b (which is associated with dental remains and, thus, more securely identified). However, SK2045 is spatially placed closer to other australopithecine specimens (see main text, Figure 2), indicating that the morphology of its articular surface is ambiguous. B, CVA of the same hominin radial sample removing SK2045. Differences between Homo and australopithecines are increased. OH80-11 seems thus even closer to the latter. The average amount of predictive error in each variable is reduced from 14% in A to 10% in B.

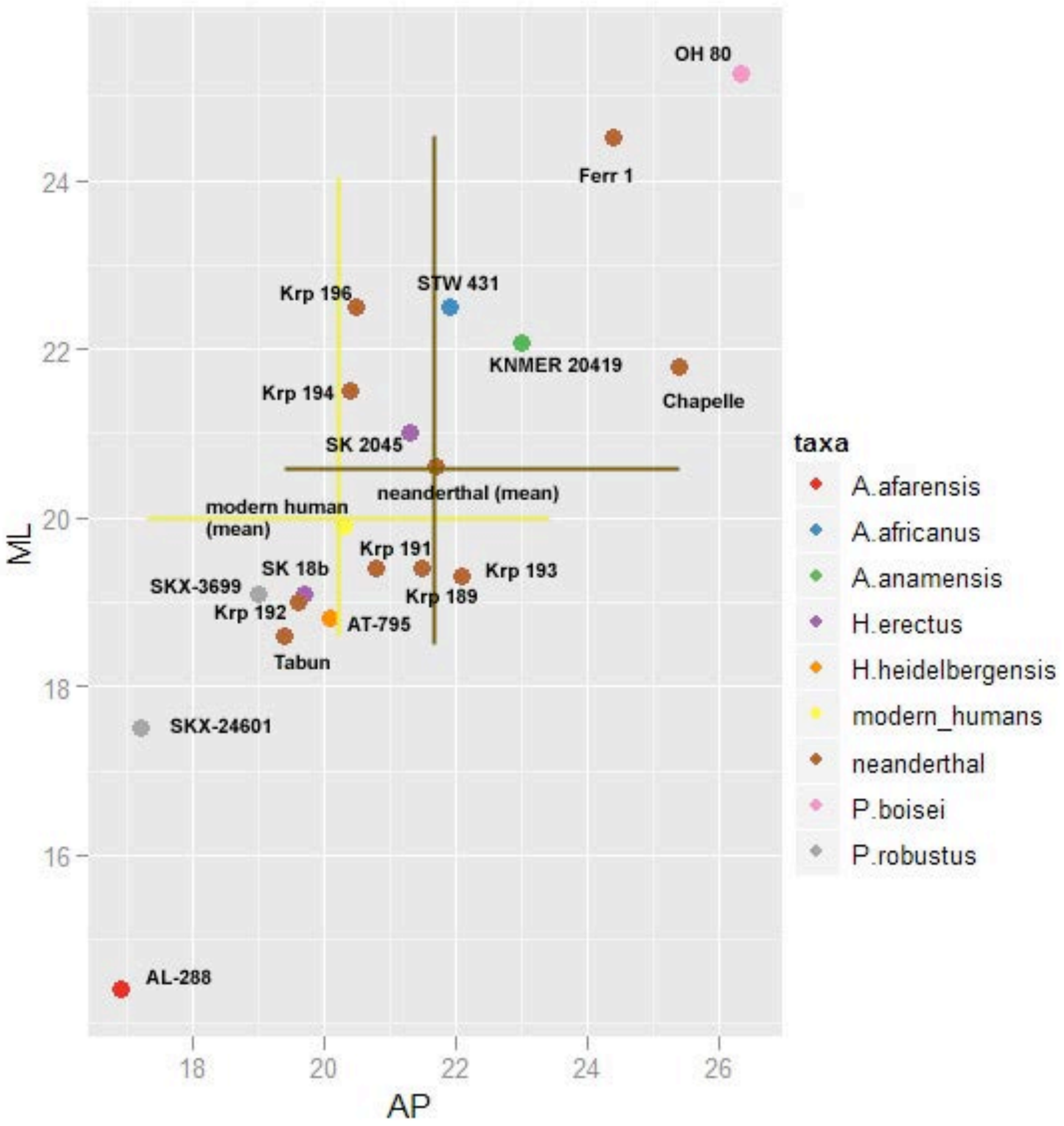


Figure S4. Dimensions (AP=anterior-posterior; ML=medial-lateral) of the proximal articular head of hominin radii of different hominins. Measurements for modern humans are from Senut [3] and for Neanderthal and Atapuerca specimens are from Carretero [4]. Abbreviations: Krp, Krapina; Ferr, Ferrasie; SKX, Swartkrans; STW, Sterkfontein; AT, Atapuerca. Bars for modern humans and Neanderthals show maxima and minima.

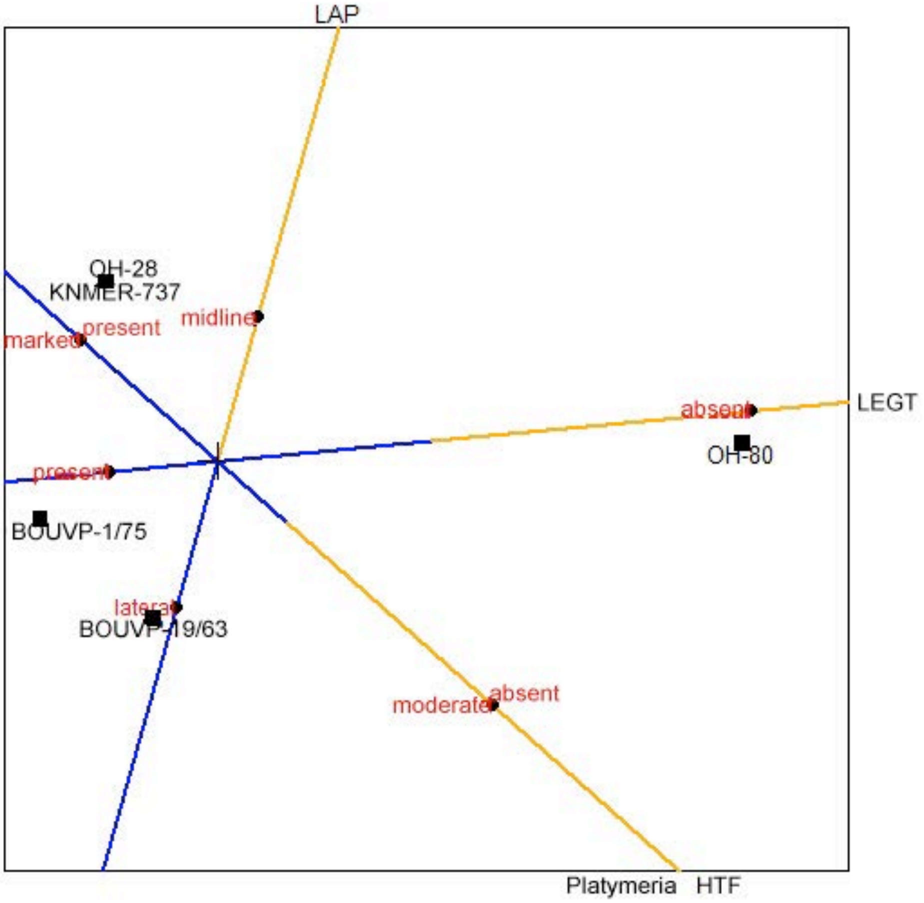


Figure S5. Multiple correspondence analysis (MCA) of OH 80-12 and the femora of *Homo erectus* . Abbreviations: HTF = hyper-trochanteric fossa; LEGT + lateral extension of the gluteal tuberosity; LAP = *linea aspera* position. *Homo erectus* data from [5].

The MCA two-dimensional solution explains 61.26% of sample variance: first dimension accounts for the largest portion (40.71% of inertia) and the second dimension accounts for the remaining variance (20.55% of inertia). The contribution of the variables (and their categories) and the cases to the result is as follows:

| | dim 1 | dim 2 |
|---|---|---|
| BOUVP-1/75 | 2.2536544 | 1.39106359 |
| BOUVP-19/63 | 0.2950215 | 10.36487076 |
| KNMER-1472 | 0.2950215 | 10.36487076 |
| KNMER-737 | 0.8828377 | 13.20852532 |
| OH-28 | 0.8828377 | 13.20852532 |
| OH80 | 19.9516239 | 0.12224147 |
| Paltymeria marked | 3.8485911 | 2.05896881 |
| Platymeria moderate | 7.6971823 | 4.11793762 |
| LAP_lateral | 2.2317795 | 19.34635521 |
| LAP_midline | 2.2317795 | 19.34635521 |
| LEGT_absent | 19.9516239 | 0.12224147 |
| LEGT_present | 3.9903248 | 0.02444829 |
| HTF_absent | 7.6971823 | 4.11793762 |
| HTF_present | 3.8485911 | 2.05896881 |

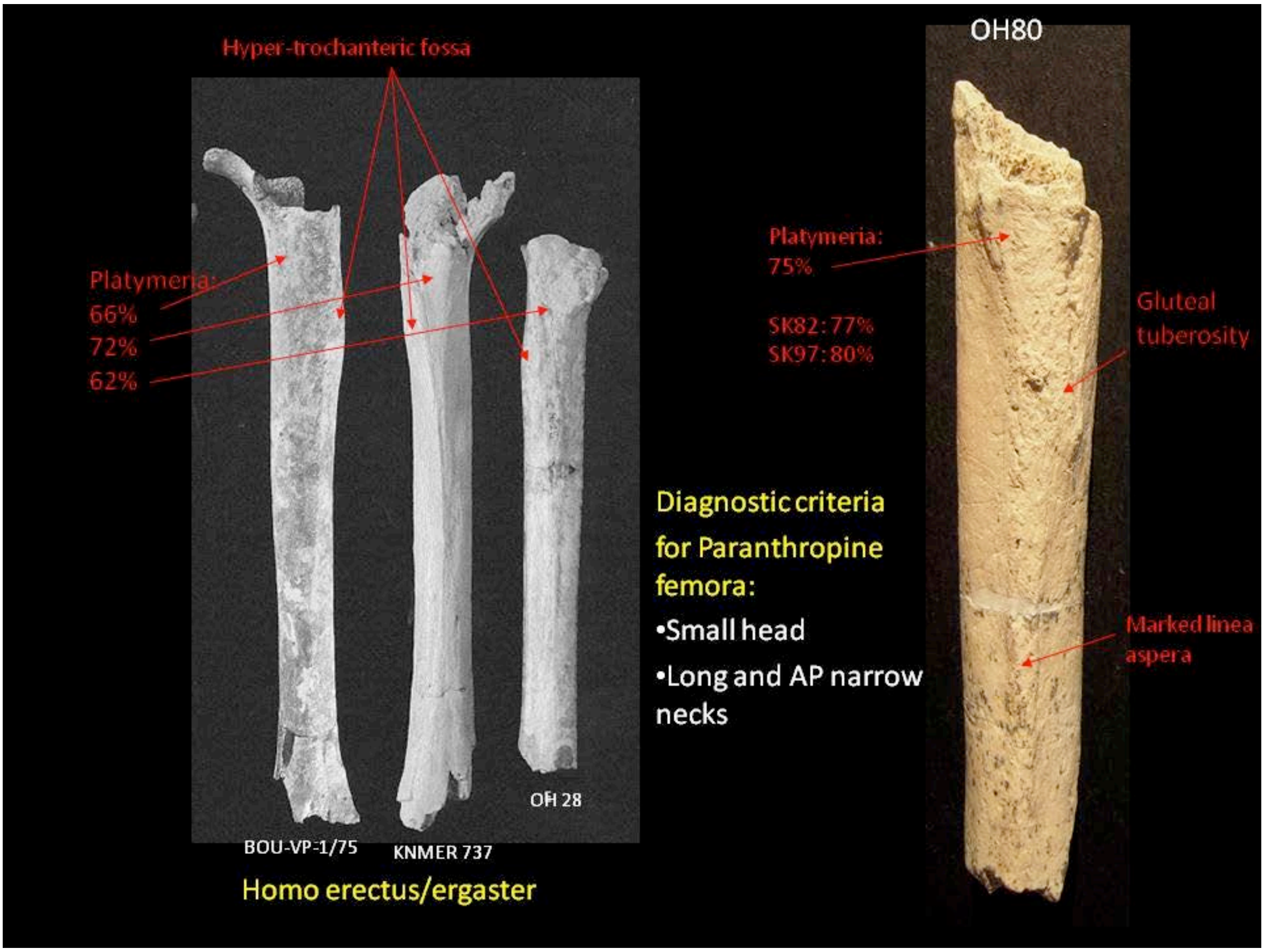


Figure S6. Comparison of OH 80-12 (right) and three representative femora of *Homo erectus* [5] , illustrating different expressions of taxonomically diagnostic criteria. Note OH 80-12's lack of a hyper-trochanteric fossa and its more medially placed *linea aspera*, which is also more robust than those of *H. erectus*.

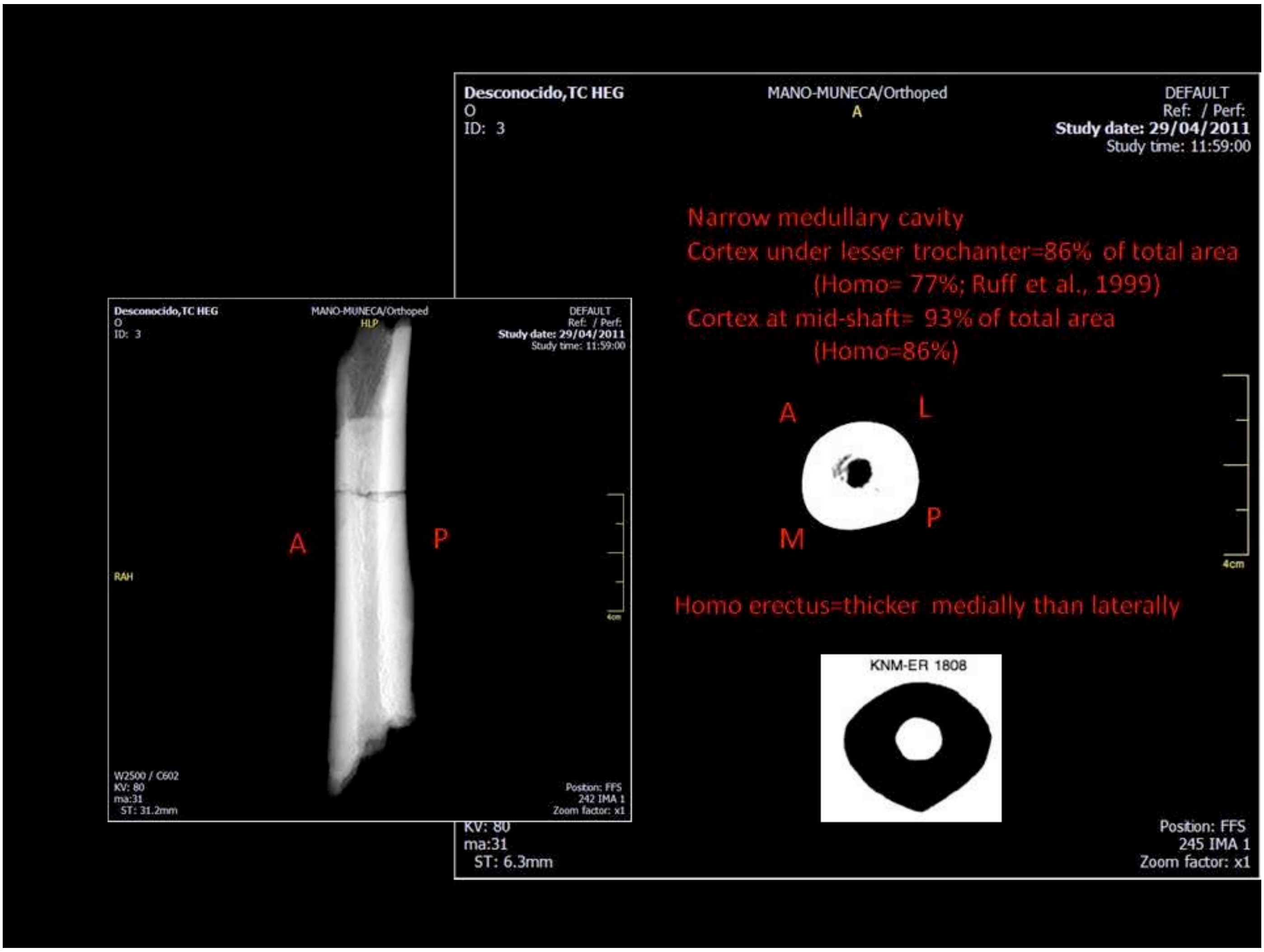


Figure 7. CT-scan image of the AP section of OH 80-12 (left) and of diaphysis section (center, right), illustrating the robust thickness of the diaphysis and thickness distribution (second moment of area) according to orientation: A = anterior; P = posterior; L = lateral; M = medial. The femur section is oriented distally (upper) to proximally (lower). The diaphysis section (center,right) is taken at the mid-diaphysis, coinciding with the natural fracture as seen on the left scan. A diaphysis section of a the KNM- ER 1808 *Homo erectus* femur is overlaid (lower right) to show differences. Note the proportionally smaller medullary cavity and more thicker cortex of OH 80-12.

**Tibia**

OH 80-13 is a portion of the well-preserved medial aspect of a tibia midshaft fragment (92.1 mm superoinferiorly). The cortex of OH 80-13 is marred by random sedimentary abrasion and also preserves two overlapping micronotches along a green spiral fracture plane, suggesting it was broken when still fresh. Like modern human tibiae, the fossil shows fine superoinferiorly trending periosteal striae, has a thick cortex (7.9 mm) and a small medullary cavity packed with trabeculae (Figure S8). The robusticity of OH 80-13 accords with the ruggedness of the other OH 80 postcranial fossils. Based on this correspondence and on the principle of parsimony, we allocate the tibia to the same individual of *P. boisei* as presumably contributed its humerus, radius and femur to the BK assemblage.

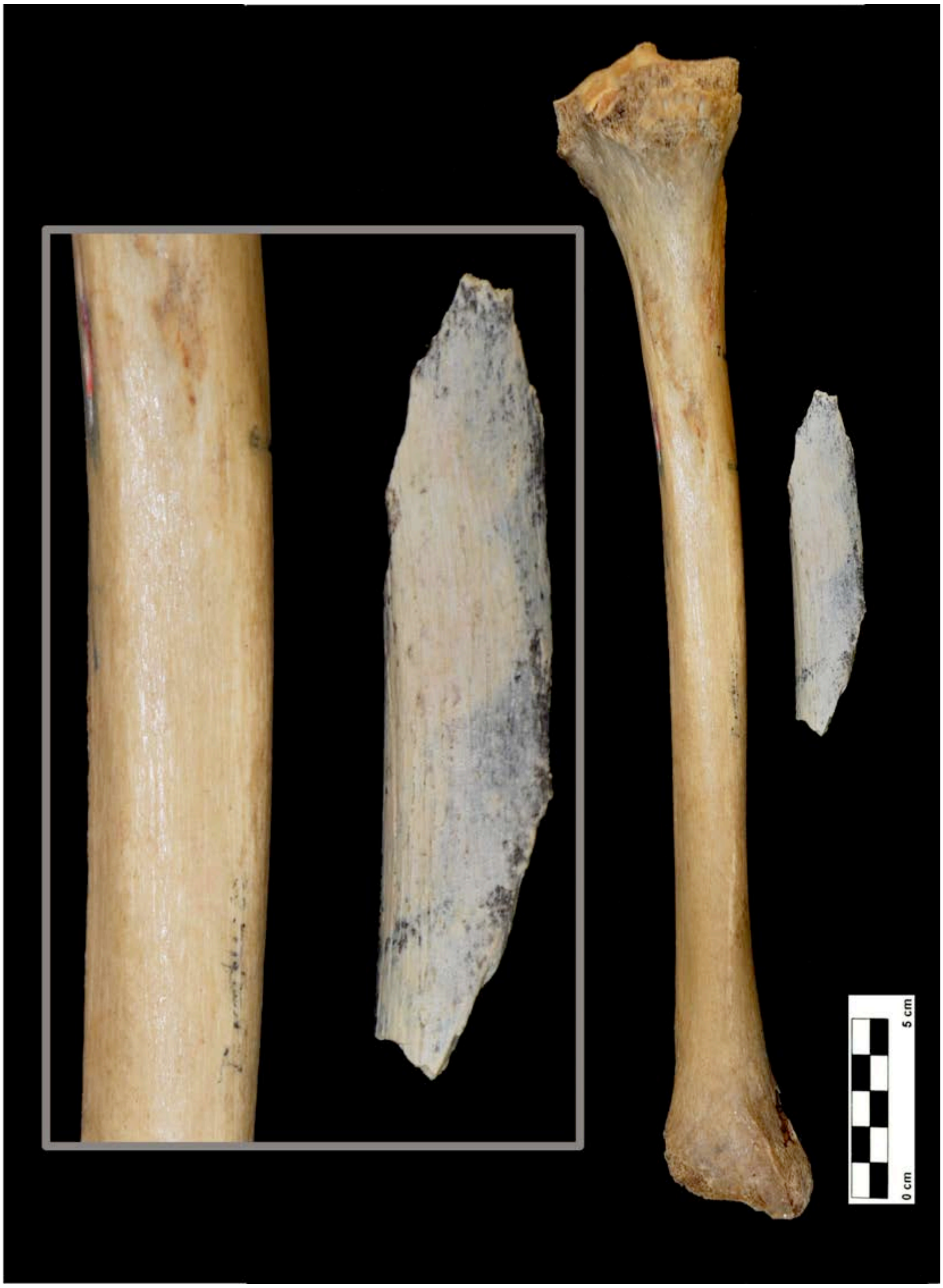


Figure S8. OH 80-13 (tibial shaft) compared to a modern human tibia. Image by E. Organista and JL Heaton.

## THE GEOLOGICAL CONTEXT OF BK

The eastern margin of the Serengeti Plain, northern Tanzania, was filled during the late Pliocene and early Pleistocene with volcanoclastic, lacustrine, alluvial, fluvial and aeolian deposits. Sedimentation spread throughout the basaltic plain and the surrounding large inselbergs of metamorphic rocks. The Olduvai River created an incision spanning from the Olbalbal depression, within a fault graben, up to Lake Ndutu lake (47 km eastward), forming the Olduvai Main Gorge, exposed throughout the whole sedimentary sequence (~100 m). The Side Gorge was developed by the Kelogi River, which drains the northwest slope of Lemagrut volcano and joints the Main Gorge 9 km upstream of Olbalbal. The sequence exposed in both gorges was defined by Reck [6] and further developed by Hay [7] into six beds. Stratigraphic correlations are based on numerous widespread tuffs represented along the whole gorge, as well as sedimentologic and stratigraphic properties of strata. Several of the Olduvai tuffs have been dated radioisotopically, and there is good age control for the fossils contained within the strata.

The BK (Bell´s Korongo) site is in the Side Gorge, 3 km upstream from the junction with the Main Gorge. Stratigraphically, it is near the top of Bed II, overlying tuff IID, which was initially dated to 1.2 Ma [8,9]. At BK, Bed II is mainly represented by alluvial and fluvial deposits that conform a facies association of medial to distal fan zones and floodplains (Figure S9). Both environments show low energy processes, represented by fine-grained sediments (i.e., fine sands, silt and clay). Coarse sands and gravels are locally restricted as bedload in channels and crevasse-splay deposits. Most of the sedimentary record is represented by lense-like to sub-parallel units with a very low depth/width ratio and planar layers of massive silt and silty sand forming laterally continuous horizons up to 1 m thick. Tuffs are irregularly interbedded along the whole sequence.

BK is preserved in a low-energy fluvial deposit within a wide channel, which has eroded the upper part of Bed II. The shape of the channel is only visible on the right margin, showing a minimum depth of 4 m, although it looks wider and deeper towards the west. The fluvial deposit is composed of at least four sedimentary units, with an overall thinning section towards the upper part of the sequence. The first two units show a gently undulating shape, inclined to the west and with decreasing thickness towards the margin of the palaeo-channel. Every unit is composed of several layers of silt and very fine sand, 30-40 cm thick and occasionally lens-shaped. Bed contacts are sharp and in some parts erosive and discontinuous. Load structures are frequent and well-identified in the contact between the clayey top layer and the sandy basefloor. Overlying Units 1 and 2, Units 3 and 4 fill the topographic depression of the channel and extend over the lateral bank hundreds of meters.  Carbonates in the top of every layer are more frequent than in the underlying units. Cemented carbonate is the most usual type documented, but hard-pans and calcretes are also present. Such post-sedimentary features point to soil development during prolonged phases of subaerial exposure. The higher in the fill sequence, the more intense the presence of carbonate.

Sedimentary Unit 1 contains the archaeological levels 3, 4 and 5. Archaeological levels 1 and 2 are within Unit 2 [9] (Figure S9). Level 4 is well represented along the whole area exposed through excavation, with an average thickness of 30 cm. The basefloor contains a locally low density bed-load, composed of carbonate aggregates, mud-clasts and few coarse sands. It infills a small scale and non-continuous

depression. However, neither erosional nor current structures (i.e., marks, ripples, cross stratification, etc.) have been identified, implying a low velocity sedimentation process. In rapid transition, the sedimentation continues with massive silt and silt-clayey, representing low stages of individual flood events.

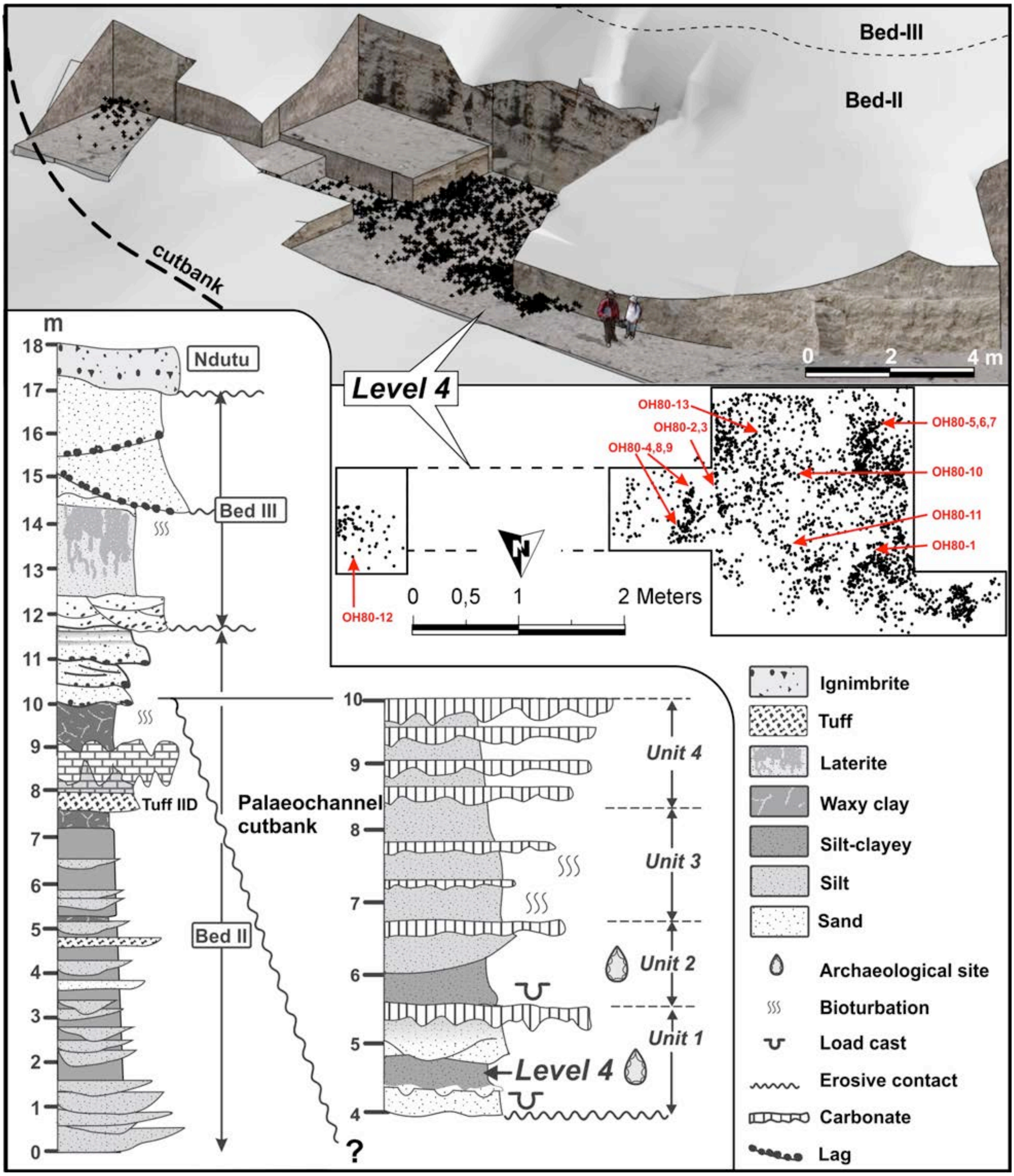


Figure S9. Stratigraphic sequence of Upper Bed II, Olduvai Gorge, and location of the BK site (right), with isometric reconstruction showing level 4 and a map with the distribution of materials and location of each of the excavated hominin fossils.

**TUFF IID**

Tuff IID is the most widespread marker in Bed II. It is reworked, discontinuous and lithologically variable in nearly the whole gorge, but it can be found relatively well preserved in localities 80 (RHC), 14 (JK), 88 (MNK), 7a (CK) and 40b (MCK) [7]. It is a trachytic tuff, generally 60 cm to 150 cm thick. The lower half of the tuff is commonly laminated, and the upper half is massive and rootmarked. It is pale gray where fresh and yellow or reddish-brown where altered to zeolites. In the Side Gorge, it is generally represented by 15 to 30 cm of tuffaceous claystone and sandtones. Tuff IID is fine- to medium-grained and is about 90% vitroclasts, principally pumice, which rarely exceed 1 mm in diameter. Most of the glass is trachytic (n= 1.520), but mafic shards are found in a few samples in the Main Gorge and may locally equal or exceed the trachytic vitroclasts in the Side Gorge. Anorthoclase, hornblende, augite, and rare nepheline suggest a phonolitic trachyte composition [7].

Stratigraphically, Tuff IID is near the top of Bed-II [7,8]. Four new samples have been collected in localities 80, 14, 40b and 94 (Figure S10).

*Locality 80 (RHC)*

Locality 80 is near the 5$^{th}$ fault, in the left margin of the Main Gorge. There is a large exposure of Beds I, II and III-IV (25, 23 and 16 m thick, respectively). RHC is the only place where Tuff IID was identified *in situ*, as a primary tuff [7]. The best outcrop extends 3.5 m underneath the top of Bed-II, which appears in a 15 m high cliff (Figure S11). Despite the good preservation, tuff IID is is a dissected layer composed by a massive tuffaceous pale yellow (5y 7/4) sandstone spanning 40 cm of thickness.

*Locality 14 (JK)*

Locality 14 is on the left margin of the main gorge, between the 4$^{th}$ and 3$^{rd}$ faults. A total section of 80 m is exposed, comprising Bed I (25 m), Bed II (26m), Bed III (14m), Bed IV (15m), Masek (1 m) and Ndutu (1 m). Here, tuff IID is a siliceous earthy siltstone, 60-70 cm thick and pale yellow (5y 7/3). The lower half is laminated and the upper half is massive and root-marked. It is located 3 m bellow the top of Bed II (Figure S11).

*Locality 40b (MCK)*

Locality 40b is on the right margin of the main gorge, close to the 4$^{th}$ fault. It shows a total section of 42 m that comprises Bed I (12m), Bed II (20 m), Bed III and IV (10m). Tuff IID is on top of Bed II, just 2.5 m below the erosive contact of Bed III. In this case, it is a complex tuff, composed by three distinctive layers (Figure S11). The lower one is preserved in the form of surge. It is 30 cm thick, silty to clayey, well laminated and light brownish grey (2.5y 6/2). The middle one is a silty tuff, 50 cm thick and the upper one is an airfall laminated tuff, pale yellow and 12 cm thick.

*Locality 94 (BK)*

Tuff IID in the Side Gorge is thin, discontinuous, and contaminated by claystone pellets and detrital sand. In this area, it probably was deposited on mudflats and reworked by the wind [7]. In locality 94, this tuff can be identified at 20 m of the BK archaeological site, as a siliceous earthy siltstone, pale yellow (5Y 8/3) and massive. At this point, it has a total thickness of 50 cm (Figure S11). Due to intensive bioturbation, it is porous and root-marked (charophytes). Stratigraphically, tuff IID is 4 m bellow the top of Bed II, and can be followed discontinuously hundreds of meters up and downstream in the Side Gorge.

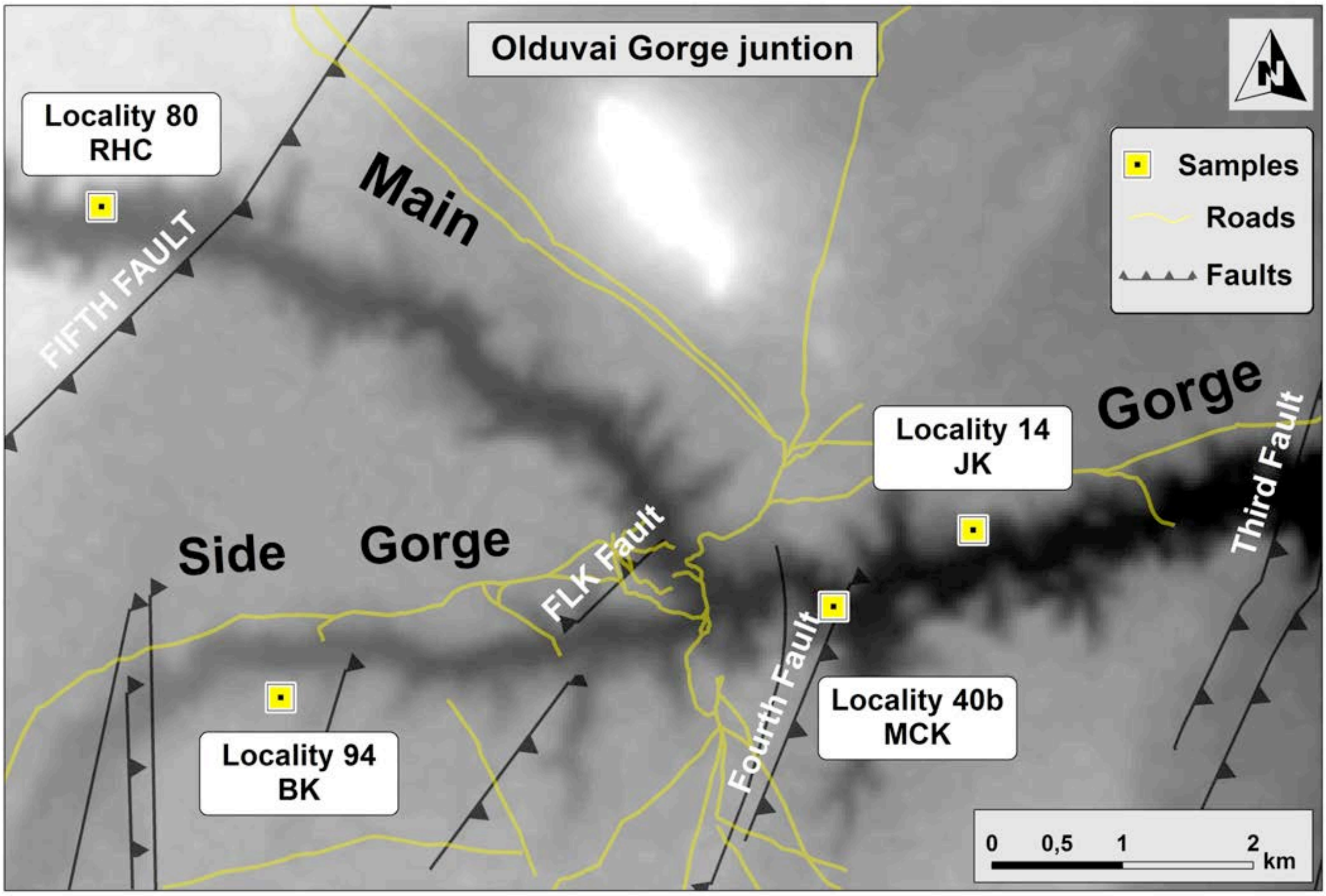


Figure S10. Map of Olduvai Gorge showing the localities where the tuff IID samples were collected.

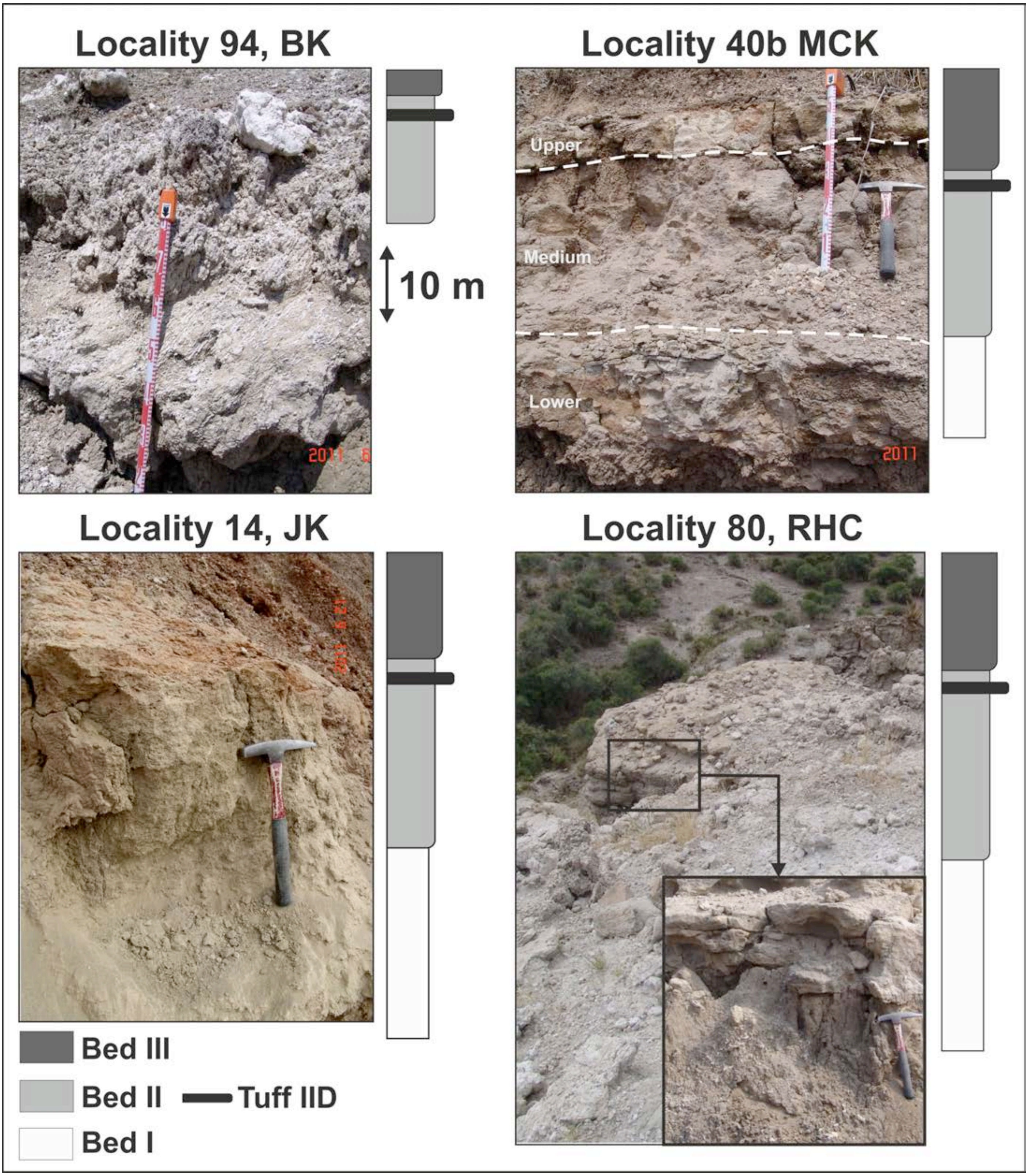

Figure S11. Photographs of Tuff IID in each of the localities and stratigraphic position of the tuff at each of them

## AR/AR METHODOLOGY AND RESULTS

*Methods*

Samples were disaggregated gently using a mortar and pestle. Sanidine was handpicked under a binocular microscope using the methods of Hyneck et al. [10] to ensure a pure separate. After leaching in dilute HF and rinsing in de-ionised water and methanol, the grains were parcelled into Cu packets and positioned within an Al holder for irradiation. International standard Alder Creek Tuff sanidine (ACs, 1.193 ± 0.001 Ma; [11]), a secondary standard referenced against the Fish Canyon age (FCs) (28.02 ± 0.16 Ma) of Renne et al. [12], was loaded adjacent to the samples of unknown age. Samples were irradiated for 120 minutes in the Cd-lined CLICIT facility of the OSU TRIGA reactor. ACs grains ($n$ = 60) were analysed by total fusion with a focused $CO_2$ laser. The J-parameter was determined to a precision of c. 0.1 %.

Single grains (100-200 µm) of unknowns (note crystals were exceptionally small) were loaded into a Cu planchette in an ultra-high vacuum laser cell with a doubly pumped ZnSe window. Using a $CO_2$ laser, the sanidine crystals were degassed at low temperature and subsequently fused. Radiogenic $^{40}$Ar ($^{40}$Ar*) yields were thus improved from 50-60% to typically greater than 80%; no $^{39}$Ar was liberated. All gas fractions were subjected to 180 s of purification with two SAES GP50 getters (one at room temperature the other at 450 ˚C) and a cold finger maintained at -95.5 ˚C using a mixture of dry ice ($CO_{2[s]}$) and acetone. Argon isotope ratios (i.e., ion beam intensities) were measured using a MAP 215-50 mass spectrometer in peak jumping mode. The mass spectrometer has a measured sensitivity of $1.13 \times 10^{-13}$ moles/volt. Both the extraction and cleanup processes were automated, as were the mass spectrometer peak jumping routines and data acquisition. Backgrounds were measured after every two analyses of unknowns and average backgrounds ± standard deviation from the entire run sequence were used to correct raw isotope measurements of unknowns. Mass discrimination was monitored by analysis of air pipettes after every five analyses. Backgrounds and discrimination (D) are reported in the excel spreadsheet containing the raw $^{40}$Ar/$^{39}$Ar data. The Ar isotope data were corrected for backgrounds, mass discrimination, and reactor-produced nuclides and processed using standard data reduction protocols. The decay constants of Steiger & Jäger [13] and atmospheric argon ratios of Lee et al. [14], the latter independently verified by Mark et al. [15], were employed.

The BGC software *MassSpec* was used for data regression. Data are displayed on ideograms and isotope correlation plots (inverse isochron plots). The standard error on the mean was determined for all samples that displayed a Gaussian (normal) distribution with a MSWD < 1. A data filter was used to screen the single crystal Ar/Ar ages for xenocrysts - any Ar/Ar age > 1.5 Median Absolute Deviations (nMADs) from the weighted mean was rejected from the age calculations. Data were not rejected on the basis of %$^{40}$Ar*.

The Ar/Ar method is a relative dating technique with all ages referenced back to a standard of known age. The ACs age of Nomade et al. [11] is determined relative to FCs with an age of 28.02 ± 0.16 Ma [12]. More recently there have been a series of updated ages suggested for FCs (16-19). These calibrations have reduced systematic uncertainties in the Ar/Ar system from *c*. 2.5 to < 0.25 %. We have recalculated our Ar/Ar ages using the optimisation model of Renne et al. [18]. Ar/Ar age uncertainties include full error propagation and these are what are reported in the main text. All raw Ar/Ar data can be located in the accompanying excel spreadsheet (Ar/Ar_data).

*Results*

Sample BK

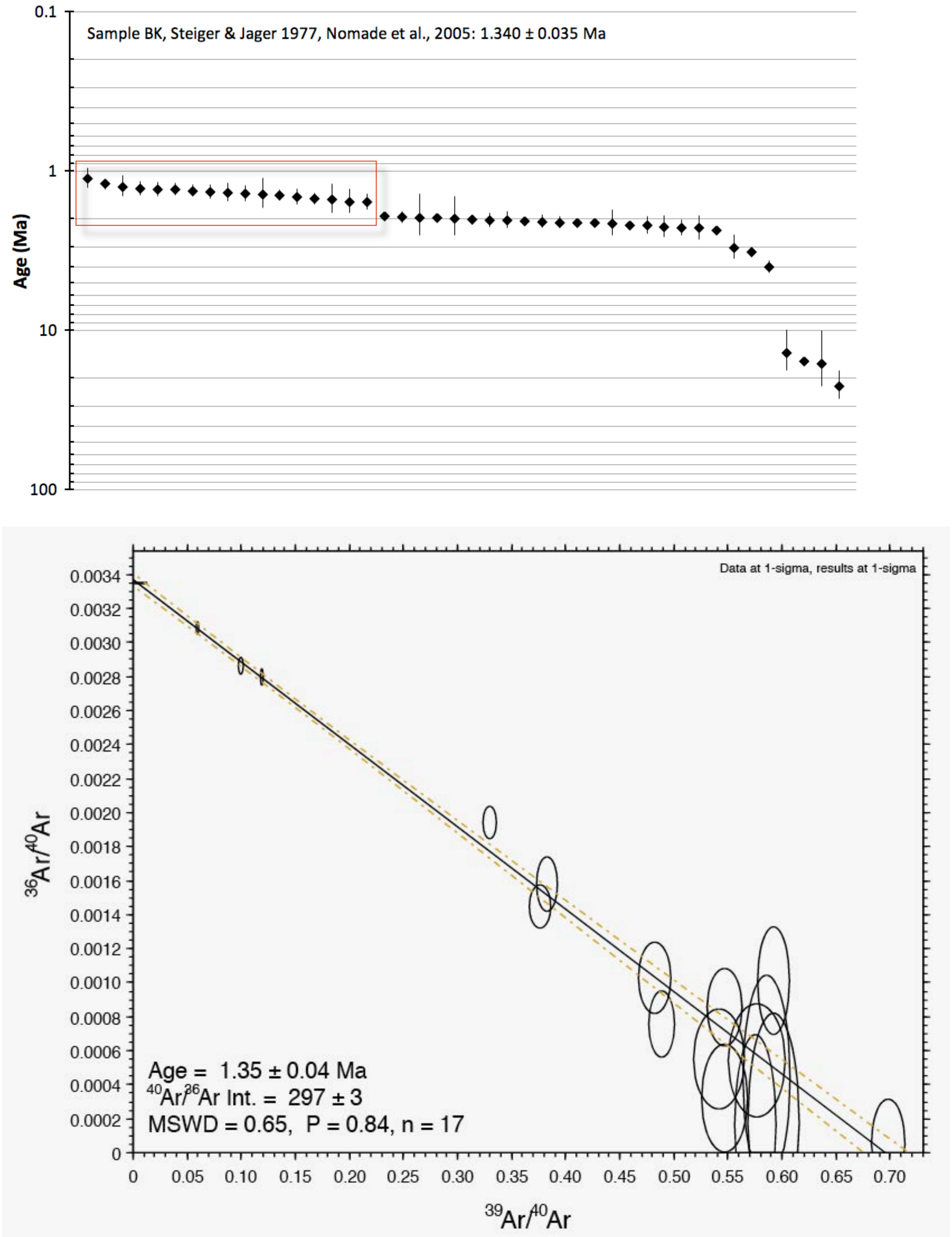


FIGURE S12. Ar/Ar results for the BK sample.

Sample RHC

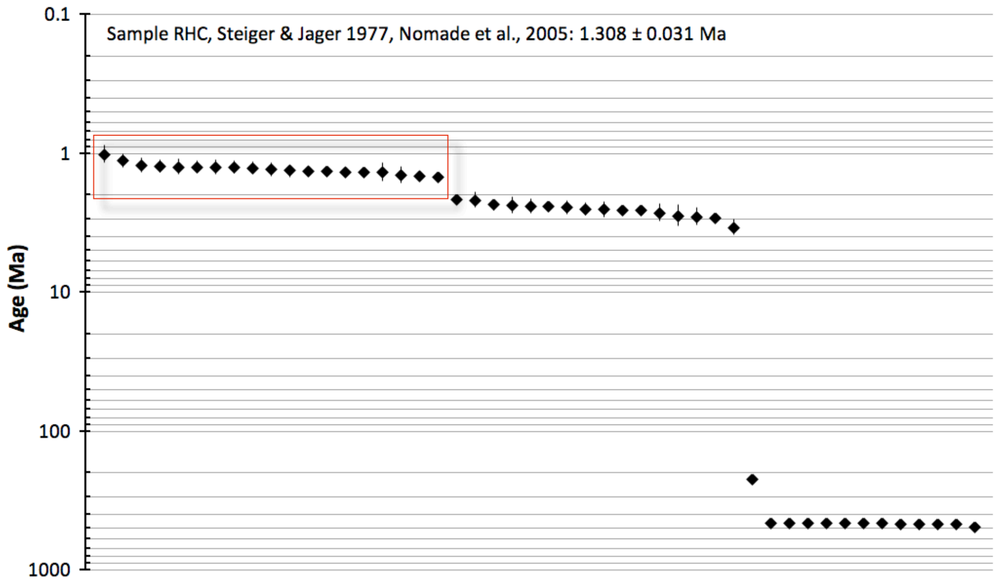


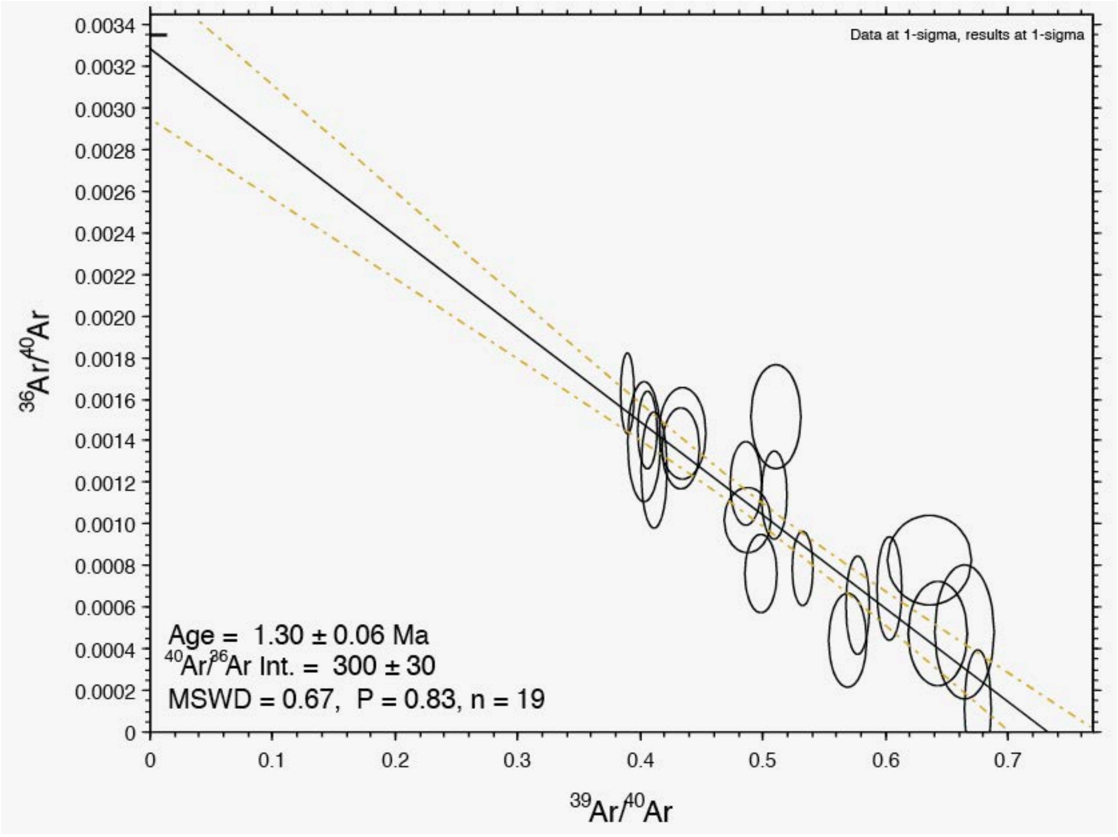


FIGURE S13. Ar/Ar results for the RHC sample.

Sample MCK

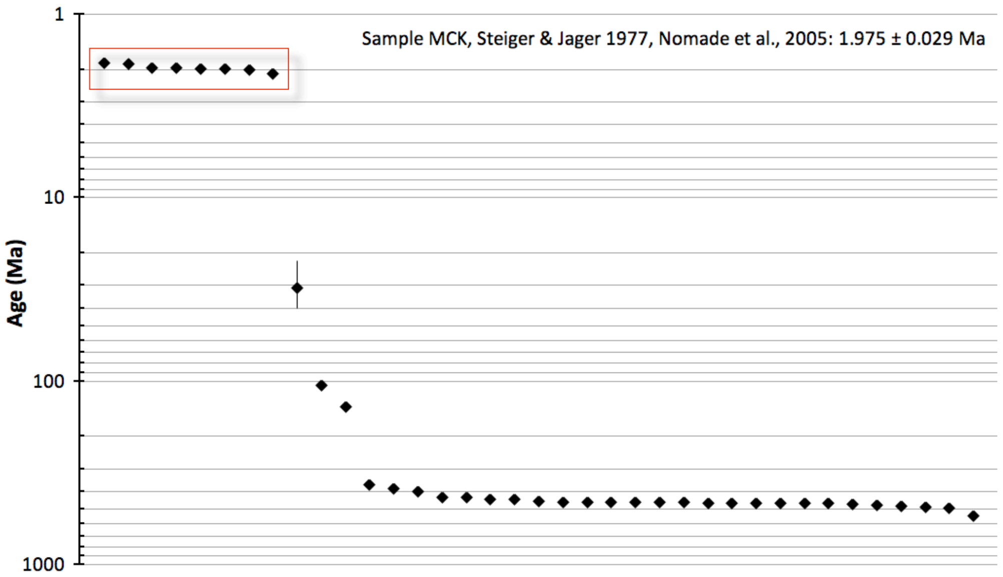


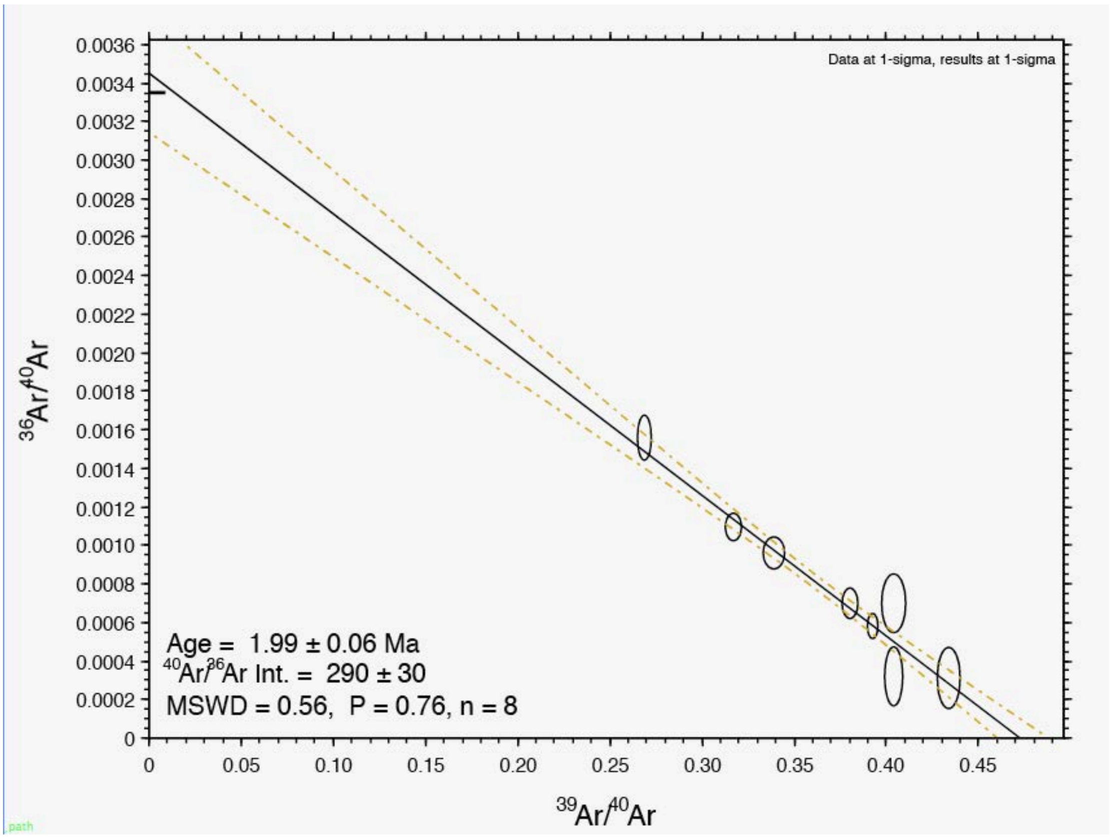


FIGURE S14. Ar/Ar results for the MCK sample.

Sample JK

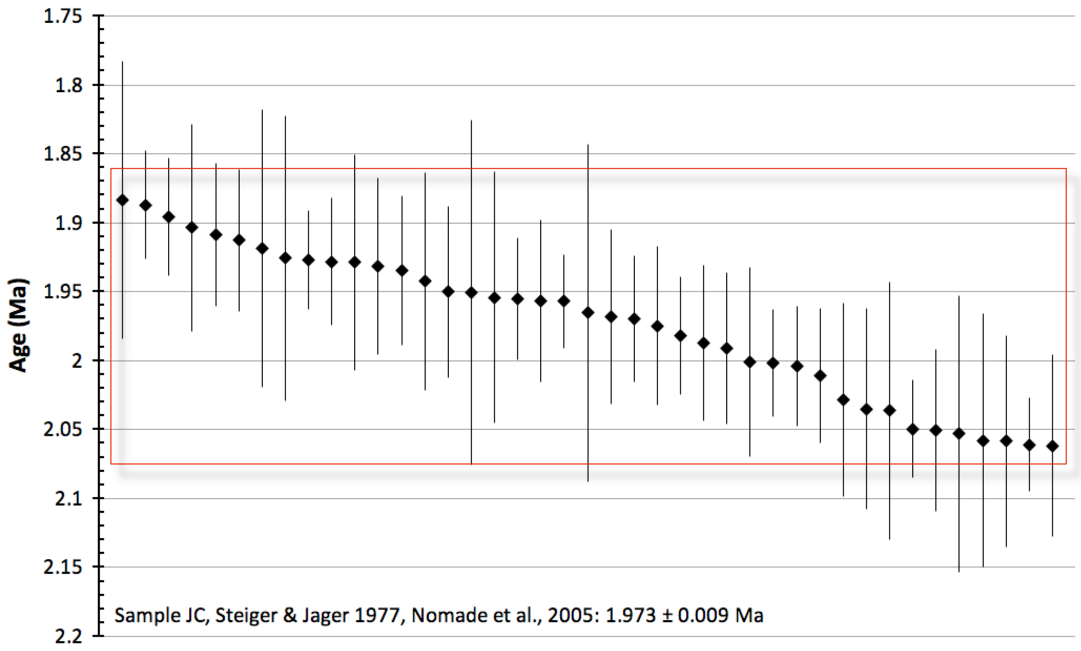


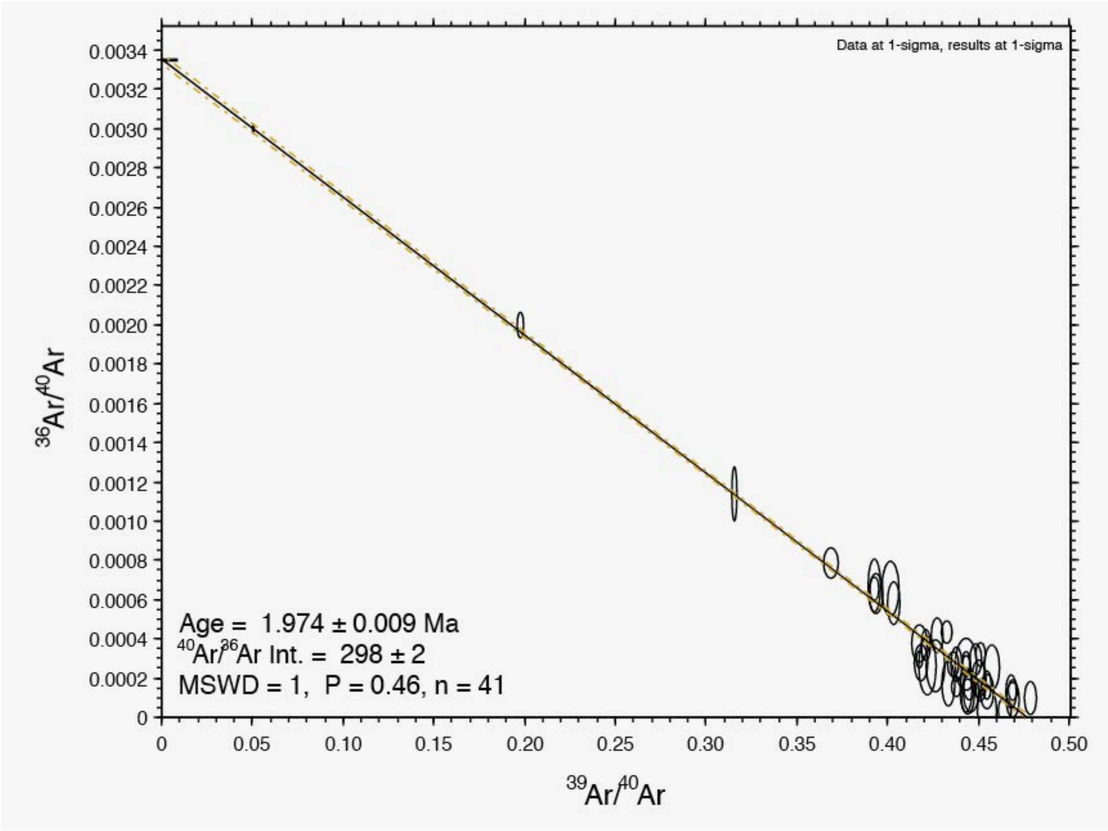


FIGURE S15. Ar/Ar results for the JK sample.

TABLE S6. Ar/Ar results are summarised in the table below and ages are calculated using two different sets of standard ages and decay constants.

| Sample | n | MSWD | Steiger & Jager 1977, Renne et al., 1998 (Ma ±1s analytical) | Renne et al., 2011 (Ma ± 1s full external precision) |
|---|---|---|---|---|
| JK | 41/41 | 0.98 | 1.973 ± 0.009 | 1.992 ± 0.009 |
| BK | 17/44 | 0.62 | 1.340 ± 0.035 | 1.353 ± 0.035 |
| MCK | 8/37 | 0.50 | 1.975 ± 0.029 | 1.995 ± 0.029 |
| RHC | 19/48 | 0.66 | 1.308 ± 0.031 | 1.321 ± 0.032 |

The Ar/Ar data show that the tuff exposed at JK and MCK do not correlate with tuff IID that is exposed at BK and RHC.

The final age of tuff IID is calculated via weighted average of the BK and RHC samples.

We report a final tuff IID age of: 1.338 ± 0.024 Ma (versus the optimisation model of [17]). It is this age that is referenced throughout the manuscript.

## TEPHROCHRONOLOGY OF TUFF IID

We attempted to characterise the tuffs using glass and mineral compositions to correlate units. Unfortunately, the glass in all the units is completely altered and not suitable for analysis. Feldspar, augite, sodic augite, hornblende and Fe-Ti oxides were found in all the units (JK, RHC, BK and MCK), and quartz, olivine, allanite and perovskite are found in some samples (see Table S7). These minerals were analysed using a JEOL-8600 electron microprobe in the Research Laboratory for Archaeology and the History of Art, University of Oxford. A 15 kV, 15 nA focussed beam was used for the analysis. The instrument was calibrated using a suite of mineral standards, which was verified using Smithsonian reference material.

None of these volcanic deposits have distinctive mineral compositions (Table S7; Figure S16). The compositions of the mineral phases span a wide range and with the variability within each deposit similar to that between deposits (Table S7). These wide compositional ranges, and the presence of phases that do not crystallise in the same magmatic system (e.g., olivine and quartz in JK and RHC) in the same sample, imply that there is xenocrystic or lithic material in the deposits, or both. This may reflect some reworking of the deposits. It is not clear how the deposits in the area have been previously correlated given that the glass is completely altered and the compositions are not distinct for any of the units. It implies that previous correlations may not be robust.

**Table S7. Mineralogy and composition of some phases in the Olduvai Gorge (Tanzania) samples**

| | K-feldspar | | | Plagioclase | | | Augite | | | | Sodic augite | Hornblende | Fe-Ti oxides | Quartz | Olivine | Perovskite | Al |
|---|---|---|---|---|---|---|---|---|---|---|---|---|---|---|---|---|---|
| | An | Or | *n* | An | Or | *n* | En | Fs | Mg♯ | *n* | | | | | | | |
| RHC | 1-15 | 1-33 | 7 | 22-27 | 1-8 | 3 | 31-40 | 10-21 | 60-80 | 5 | | x | x | x | x | x | |
| BK | 2-18 | 0-30 | 13 | 27-45 | 4-8 | 6 | 28-45 | 8-23 | 56-86 | 17 | x | x | x | x | | x | |
| JK | 1-17 | 8-40 | 8 | 25-57 | 2-8 | 9 | 31-44 | 8-18 | 67-85 | 18 | | x | x | x | x | | |
| MCK | 0-16 | 1-53 | 26 | 24-54 | 2-7 | 12 | 30-40 | 10-21 | 60-80 | 12 | x | x | x | | x | x | |

x = present. *n*= number of analyses. See Supplementary Material for all the raw data.

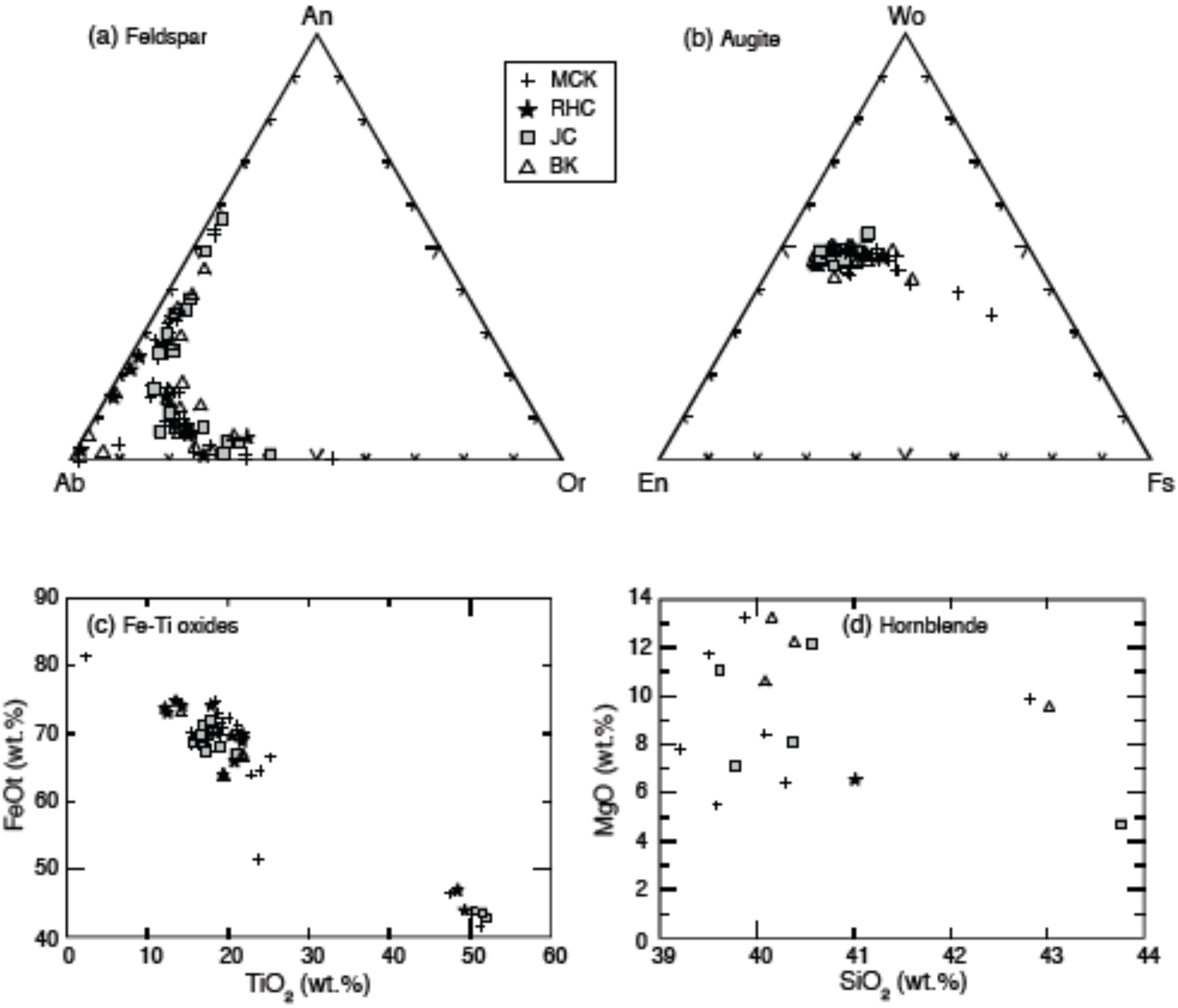


Figure S16. Compositions of mineral phases in the Olduvai tuff samples. (a) Ab-rich anorthoclase and plagioclase feldspar compositions. (b) Augite and sodic augite compositions. (c) Spinel and rhombohedral (Ti-rich) Fe-Ti oxide compositions. (d) Hornblende compositions.

**REFERENCES**


1. Susman RL, de Ruiter D, Brain CK (2001) Recently identified postcranial remains of *Paranthropus* and early *Homo* from Swartkrans Cave, South Africa. *J Hum Evol* 41:607-629.
2. Patel B (2005) The hominoid proximal radius: re-interpreting locomotor behaviors in early hominins. *J Hum Evol* 48:415-432.
3. Senut B (1981) *L´humerus et ses articulations chez les hominids Plio-Pleistocènes*. (Cahiers de

Paleóntologie. Editions du CNRS, Paris).
4. Carretero JM (1994) Estudio del esqueleto de las dos contiras y el miembro superior de los homínidos de la Sima de los Huesos, Sierra de Atapuerca, Burgos. PhD dissertation, Complutense University, Madrid.
5. Gilbert WH, Asfaw B (2009) Homo erectus: *Pleistocene Evidence from the Middle Awash, Ethiopia* (University of California Press, Berkeley).
6. Reck H (1951) A preliminary survey of the tectonics and stratigraphy of Olduvai. In *Olduvai Gorge, Vol. I*, ed Leakey LSB (Cambridge University Press, London).
7. Hay RL ( 1976) *Geology of the Olduvai Gorge*. University of California Press, Berkeley.
8. Leakey MD (1971) *Olduvai Gorge, Vol. III: Excavations in Bed I and II, 1960-63* (Cambridge University Press, Cambridge).
9. Domínguez-Rodrigo M, Mabulla A, Bunn, HT, Diez-Martín F, Barba R, Egeland CP, Espílez E, Egeland A, Yravedra J, Sánchez P (2009) Unravelling hominid behavior at another anthropogenic site from Olduvai Gorge (Tanzania): new archaeological and taphonomic research at BK, Upper Bed II. *J Hum Evol* 57:260-283.
10. Hynek SA, Brown FH, Fernandez D (2010) A rapid method for hand picking potassium-rich feldspar from silicic tephra. *Quaternary Geochronology* 6: 285-288.
11. Nomade S, Renne PR, Vogel N, Deino AL, Sharp WD, Becker T, Jaouni AR, Mundil R (2005) Alder Creek sanidine (ACs-2): a Quaternary $^{40}Ar/^{39}Ar$ dating standard tied to the Cobb Mountain geomagnetic event. *Chemical Geology* 218: 315-338.
12. Renne PR, Swisher CC, Deino AL, Karner DB, Owens TL, DePaolo D. (1998) Intercalibration of standards, absolute ages and uncertainties in $^{40}Ar/^{39}Ar$ dating. *Chemical Geology* 145: 117– 152.
13. Steiger RH, Jäger E (1977) Subcommission on geochronology: convention on the use of decay constants in geo- and cosmochronology. *Earth and Planetary Science Letters* 36:359-362.
14. Lee J-L, Marti K, Severinghaus J, Kawamura K. Yoo H-S, Lee J, Kim JS. 2006. A redetermination of the isotopic abundances of atmospheric Ar. *Geochimica et Cosmochicima Acta* 70: 4507–4512.
15. Mark DF, Stuart FM, de Podesta M (in press) New high-precision measurements of the isotopic composition of atmospheric argon. *Geochimica et Cosmochimica Acta*, doi:10.1016/j.gca.2011.09.042
16. Kuiper K., Deino A, Hilgen FJ, Krijgsman W, Renne P, Wijbrans J (2008) Synchronizing Rock Clocks of Earth History. *Science* 320:500-504.
17. Renne PR, Mundil R, Balco G, Min K, Ludwig K (2010) Joint determination of $^{40}K$ decay constants and $^{40}Ar^*/^{40}K$ for the Fish Canyon sanidine standard, and improved accuracy for $^{40}Ar/^{39}Ar$ geochronology. *Geochimica et Cosmochimica Acta* 74: 5349–5367
18. Renne PR, Balco G, Ludwig K, Mundil R, Min K, (2011) Response to the comment by W.H. Schwarz et al. on “Joint determination of $^{40}K$ decay constants and $^{40}Ar^*/^{40}K$ for the Fish Canyon sanidine standard, and improved accuracy for $^{40}Ar/^{39}Ar$ geochronology” by P.R. Renne et al., 2010. *Geochimica et Cosmochimica Acta* 75: 5097-5100.
19. Rivera T, Storey M, Kuiper K, Pälike H, 2011. Towards an integrated geomagnetic polarity reversal timescale for the Pleistocene. AGU Fall meeting, San Francisco, USA.
